# Metal binding and interdomain thermodynamics of mammalian metallothionein-3: enthalpically favoured Cu^+^ supplants entropically favoured Zn^2+^ to form Cu_4_^+^ clusters under physiological conditions†

**DOI:** 10.1039/d2sc00676f

**Published:** 2022-04-04

**Authors:** Matthew R. Mehlenbacher, Rahma Elsiesy, Rabina Lakha, Rhiza Lyne E. Villones, Marina Orman, Christina L. Vizcarra, Gabriele Meloni, Dean E. Wilcox, Rachel N. Austin

**Affiliations:** Department of Chemistry, Dartmouth College Hanover NH 03755 USA; Department of Chemistry, Barnard College of Columbia University New York NY 10027 USA raustin@barnard.edu; Department of Chemistry and Biochemistry, University of Texas at Dallas Richardson TX 75080 USA

## Abstract

Metallothioneins (MTs) are a ubiquitous class of small metal-binding proteins involved in metal homeostasis and detoxification. While known for their high affinity for d^10^ metal ions, there is a surprising dearth of thermodynamic data on metals binding to MTs. In this study, Zn^2+^ and Cu^+^ binding to mammalian metallothionein-3 (MT-3) were quantified at pH 7.4 by isothermal titration calorimetry (ITC). Zn^2+^ binding was measured by chelation titrations of Zn_7_MT-3, while Cu^+^ binding was measured by Zn^2+^ displacement from Zn_7_MT-3 with competition from glutathione (GSH). Titrations in multiple buffers enabled a detailed analysis that yielded condition-independent values for the association constant (*K*) and the change in enthalpy (Δ*H*) and entropy (Δ*S*) for these metal ions binding to MT-3. Zn^2+^ was also chelated from the individual α and β domains of MT-3 to quantify the thermodynamics of inter-domain interactions in metal binding. Comparative titrations of Zn_7_MT-2 with Cu^+^ revealed that both MT isoforms have similar Cu^+^ affinities and binding thermodynamics, indicating that Δ*H* and Δ*S* are determined primarily by the conserved Cys residues. Inductively coupled plasma mass spectrometry (ICP-MS) analysis and low temperature luminescence measurements of Cu-replete samples showed that both proteins form two Cu_4_^+^–thiolate clusters when Cu^+^ displaces Zn^2+^ under physiological conditions. Comparison of the Zn^2+^ and Cu^+^ binding thermodynamics reveal that enthalpically-favoured Cu^+^, which forms Cu_4_^+^–thiolate clusters, displaces the entropically-favoured Zn^2+^. These results provide a detailed thermodynamic analysis of d^10^ metal binding to these thiolate-rich proteins and quantitative support for, as well as molecular insight into, the role that MT-3 plays in the neuronal chemistry of copper.

## Abstract 2

Metallothioneins (MTs) are a ubiquitous class of small metal-binding proteins involved in metal homeostasis and detoxification.

## Graphical abstract

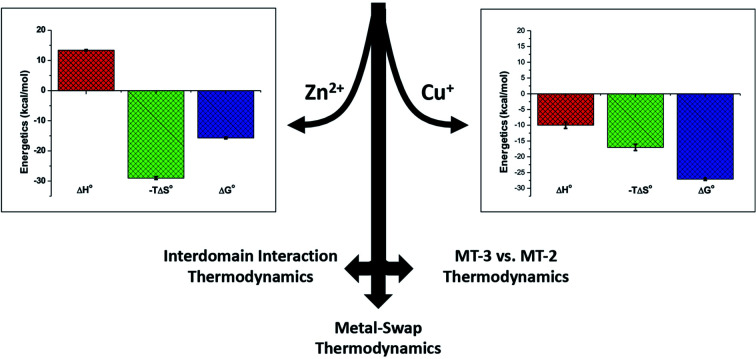

## Introduction

**Fig. 1:**
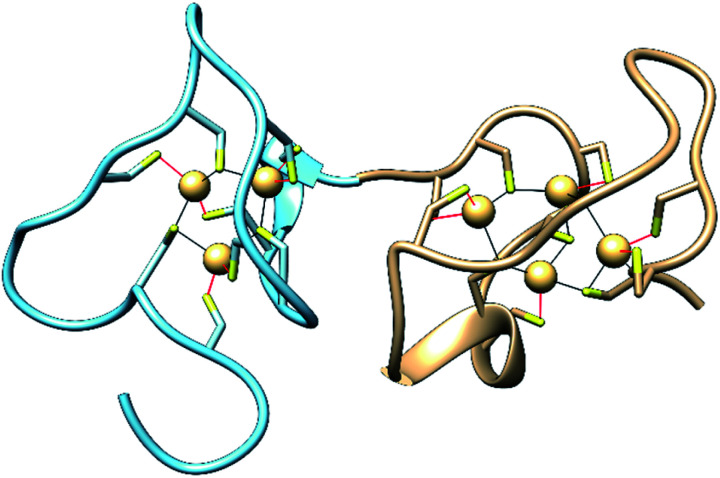
Structures of the N-terminal β-domain (blue, PDB: 1MHU), containing 3 Cd^2+^ ions bound to 9 cysteine residues, and the C-terminal α-domain (tan, PDB: 2MHU), containing 4 Cd^2+^ ions bound to 11 cysteine residues, determined by NMR measurements,^6^ of the human MT-2 protein.

Metallothioneins (MTs) are small cysteine-rich proteins found in all domains of life, with mammals expressing four isoforms, MT-1, MT-2, MT-3 and MT-4.^1,2^ Mammalian MTs contain 61–68 amino acids, 20 of which are highly conserved cysteines that bind up to seven divalent metal ions in two metal–thiolate clusters in two distinct domains of the protein (Fig. 1).^3,4^ The structurally characterized Zn- and Cd-bound MTs feature a M_3_Cys_9_ cluster in the N-terminal β-domain and a M_4_Cys_11_ cluster in the C-terminal α-domain.^5,6^ The primary roles of MTs are to sequester toxic metal ions^7^ and to buffer the concentration of Zn^2+^ and potentially Cu^+^ ions.^4,8–12^ They can also reductively quench reactive oxygen species (ROS) and reactive nitrogen species (RNS),^7,13–17^ and they have been linked to protective roles in neurodegenerative diseases and other CNS pathologies.^7,18–25^ Nevertheless, a complete understanding of the biological roles of MTs, especially isoform-specific functions, remains elusive.^4,26–34^

Metal homeostasis in all living organisms is tightly controlled by biological molecules that bind metal ions with affinities spanning many orders of magnitude.^35–40^ The rich palette of potential ligands, coupled with the impact of concentration gradients on metal-binding thermodynamics, makes well-orchestrated metal trafficking pathways possible.^37,41–43^ Cu^+^ is the most tightly controlled essential metal ion, with one of the lowest availabilities of free ions. The biological window of free copper concentrations in eukaryotic cells has been estimated to be between 10^−21^ and 10^−18^ M.^44,45^ A large thermodynamic gradient moves Cu^+^ into copper-requiring biomolecules.^37,41,43,46,47^

Metallothioneins are implicated in metal homeostasis, which makes it important to understand the metal-binding thermodynamics of the different isoforms.^48^ MT-3 is a particularly enigmatic member of the MT family.^7,18,49–54^ First identified in brain extracts as a factor that inhibited neuronal sprouting, it was initially named “neuronal growth inhibitor factor (GIF)”^15,55,56^ and only later reclassified as a metallothionein.^55^ The MT-3 isoform is distinctive in several ways.^57^ It is expressed primarily in neuronal tissues where it is localized to specific regions of the Central Nervous System (CNS),^50,58^ its sequence has two unique features (a conserved threonine insert at position 5 followed by a pair of proline residues in the β-domain (residues 7 and 9) and an acidic insert in the α-domain), and it inhibits neuronal growth.^27,29,32,49,54,57,59–67^ In contrast to MT-1 and MT-2, the expression of MT-3 is not upregulated by Zn^2+^ nor other divalent toxic metals (*e.g.* Cd^2+^), suggesting that its role in metal homeostasis is different from that of the more ubiquitously expressed MTs.^68–70^ MT-3 is, however, upregulated by hypoxia.^52,71–73^

Metallothionein-3 may modulate copper toxicity.^74–76^ Its sequence and structural properties give it the most pronounced Cu–thionein, in contrast to Zn–thionein, character of the MTs,^30,74,77^ and it is isolated from mammalian brains in a surprisingly air-stable Cu_4_^+^Zn_3–4_^2+^MT-3 form.^77–79^ MT-3 is capable of exchanging Zn^2+^ for copper that is bound to several neurologically important peptides, including amyloid β and α-synuclein, thereby eliminating harmful redox chemistry associated with Cu^2+^ in these coordination environments.^61,80,81^ The driving force for this “metal swap” is presumed to be the high affinity of MT-3 for Cu^+^, coupled with its ability to reduce Cu^2+^ to Cu^+^*via* intramolecular disulfide bond formation.^82^ MT-3 may also scavenge Cu^+^ in the reducing environment of the cell and help to maintain the low levels of free copper in neuronal cells.

In this study we have quantified the thermodynamics of Cu^+^ binding to MT-3 and MT-2, as well as the thermodynamics of Zn^2+^ binding to MT-3 and its isolated domains at physiological pH. We also provide evidence that Cu^+^ forms Cu_4_^+^–thiolate clusters that are structurally similar to those found in the β-domain of Cu_4_^+^Zn_3–4_^2+^MT-3 isolated from neuronal tissue.^78,82,83^ The thermodynamics of the competition between these two metal ions for MT-3 and domain–domain interaction in metal binding by MT, determined here, provide new insight on the molecular basis for the biological roles of these proteins.

## Materials and methods

### Reagents

Buffer salts, including MES, Bis–Tris, Tris, TAPSO and phosphate, were purchased in the highest purity available from Sigma-Aldrich, as were metal salts, dithiothreitol (DTT), 5,5′-dithio-bis-(2-nitrobenzoic acid) (DTNB), 2,2-dithiodipyridine, imidazole, β-mercaptoethanol (βME), phenylmethylsulfonyl fluoride (PMSF), reduced l-glutathione (GSH), acetonitrile (MeCN), tris(2-carboxyethyl)phosphine (TCEP), ethylenediaminetetraacetic acid (EDTA), DNAse, and diethylenetriaminepentaacetic acid (DTPA), and used without further purification. Ampicillin and isopropylthio-galactoside (IPTG) were purchased from GoldBio. HisPur cobalt resin, Ni NTA resin, Lysogeny Broth (LB) and Terrific Broth (TB) were purchased from Thermo Fisher. The HiLoad 26/600 Superdex 75 pg column, HiTrap 5 mL desalting column and HisTrap FF 5 mL affinity column were all obtained from GE Healthcare, now Cytiva. Amicon supplied the 3 kDa concentrator tubes. Recombinant His6-tagged TEV protease was expressed at 18 °C in BL21-DE3* cells and purified *via* cobalt-affinity chromatography as described below for MT-3. The only difference was an additional wash with 5 mM ATP in TALON wash buffer (50 mM Na_2_HPO_4_, 300 mM NaCl, pH 7.0, 1 mM βME). The eluted protein was dialyzed against 50 mM Tris, 250 mM NaCl, 0.5 mM TCEP, pH 8.0 for 3 h, followed by overnight dialysis into 1 : 1 glycerol-to-buffer. The sample was centrifuged at 60 000 × *g* for 10 min, flash frozen, and stored at −80 °C.

Buffer solutions for ITC measurements were prepared with Nanopure (18 MΩ) water in acid-washed glassware and subsequently treated overnight with Chelex 100® cation exchange resin (Sigma Aldrich) to ensure the absence of metal contaminants. Buffer solutions were then filtered and degassed under vacuum with stirring for at least 1 h, or until no further bubbles formed, and finally moved into an anaerobic Coy glovebox with a 95% N_2_ and 5% H_2_ environment. Chelator and metal stock solutions were prepared with oxygen-free buffer solutions and stored in the glovebox. Solutions of GSH were made fresh for each measurement. The concentration of metal stock solutions was confirmed with ITC by titrating the metal solution into a known concentration of EDTA and verifying with the binding stoichiometry, as well as confirming the known binding enthalpy. Similarly, the concentration of chelator stock solutions was confirmed with ITC by titrating a metal solution of known concentration into the chelator solution and verifying with the binding stoichiometry, as well as confirming the known binding enthalpy.

### Metallothionein-3 (MT-3) expression, purification and characterization

An Äkta Pure 25 FPLC was used for protein purification and a Coy Chamber was used for anaerobic protein manipulations. Lower speed centrifugations were performed on a Beckman Coulter Allegra X-30 R and higher speed centrifugations above 10 000 rpm were performed on a Thermo Scientific WV+ Ultra 100. Mass spectrum measurements were obtained on a Bruker ultrafleXtreme MALDI-TOF/TOF at the Columbia University mass spectrometry facility in the Chemistry Department.

Mouse MT-3 (*mus*MT3) plasmids were constructed from synthetic gene fragments that were codon-optimized for expression in *E. coli* (Integrated DNA Technologies) and a modified version of the pET15b vector. MT-3 genes were inserted in frame with an N-terminal His_6_-Green Fluorescence Protein (GFP) tag and a TEV protease site using FastCloning.^84^ Complete gene sequences are provided in Fig. S1.† BL21-DE3 cells were transformed with the His_6_-GFP-tev-*mus*MT-3 plasmid. A single colony was inoculated and grown overnight at 37 °C in LB with ampicillin (100 μg mL^−1^). In the morning, 2.8 L flasks, each containing 500 mL of TB, were inoculated with 10 mL of the overnight culture. TB cultures were then incubated at 37 °C and 250 rpm until the optical density at 600 nm (OD_600_) reached 0.7, whereupon 1 mM IPTG was added to each flask. Cultures were incubated for an additional hour at 25 °C and 250 rpm, and then 0.5 mM ZnCl_2_ was added to each flask, which was shaken for an additional 4–5 h. The MT-3-GFP cultures were centrifuged at 7500 × *g* for 15 min at 4 °C. Cell pellets were collected and resuspended into 4 mL of 50 mM sodium phosphate, 300 mM NaCl, pH 8.0, 1 mM βME per 1 g of cell pellet and either flash frozen in liquid nitrogen prior to storage at −80 °C or lysed immediately for protein isolation. Prior to cell lysis, 0.1 mM PMSF and 4 μg μL^−1^ DNAse were added to the cell suspension. Cells were either French pressed in a single pass with a Glen Mill press operating at 20 000 kPa or sonicated at 20 W for 5 s on and 10 s rest for a total of 3 min of sonication. Lysed cells were centrifuged at 40 000 × *g* for 30 min at 4 °C and the supernatant was collected.

The crude cell lysate was added to HisPur cobalt resin and nutated for 1 h at 4 °C. The resin was washed with extraction buffer (50 mM Na_2_HPO_4_, 300 mM NaCl, 1 mM βME, pH 8.0), followed by wash buffer (50 mM Na_2_HPO_4_, 300 mM NaCl, pH 7.0, 1 mM βME). His_6_-GFP-tev-MT-3 was eluted from the resin with wash buffer containing 200 mM imidazole. A PD10 column, or a 3 kDa spin-concentrator, or dialysis for 3 h, was then used to exchange the buffer of the elution fractions into PBS buffer (140 mM NaCl, 2.7 mM KCl, 10 mM Na_2_HPO_4_, 1.8 mM KH_2_PO_4_, pH 7.3, 1 mM DTT). The GFP molar extinction coefficient at 488 nm, *ε*_488_ = 56 000 M^−1^ cm^−1^, was used to determine the concentration of His_6_-GFP-tev-MT-3. Fractions were analyzed by SDS-PAGE.

To remove the His_6_-GFP tag, His_6_-TEV protease was added in a 1 : 100 protease : GFP-tev-MT-3 molar ratio. The sample tube was flushed with argon and incubated at 4 °C overnight. The sample was either loaded onto a HisTrap 5 mL FF column equilibrated with PBS buffer or rocked with pre-washed Ni-NTA resin with PBS buffer for 1 h. The flowthrough, containing cleaved MT-3, was collected from the column, concentrated in a 3 kDa spin-concentrator at 3900 × *g* at 4 °C, and further purified on a HiLoad 26/600 Superdex 75 pg size exclusion chromatography (SEC) column equilibrated with MES buffer (100 mM MES, 150 mM NaCl, 1 mM DTT, pH 6.4). Eluted fractions from the gel filtration column were analyzed by SDS-PAGE. Fractions with the desired protein were combined and concentrated using a 3 kDa spin-concentrator. MT-3 was characterized by SDS-PAGE using the monobromobimane-labeling method optimized by Meloni *et al.*^62,85^ and by MALDI-TOF mass spectrometry.

Metal ions were removed from the protein by lowering the pH to 3 with 1 M HCl in the anaerobic chamber. The resulting apo-protein was then desalted with a HiTrap desalting column that was equilibrated with 100 mM MES, 150 mM NaCl, pH 6.4, in order to separate free metal ions and exchange the protein into a buffer without DTT. The protein concentration was determined by measuring the absorption at 220 nm in 0.1 M HCl, using *ε*_220_ = 53 000 M^−1^ cm^−1^.^86^ The free thiol concentration was determined spectrophotometrically, after the reaction of MT-3 with DTNB in 25 mM Tris, 50 mM NaCl, 1 mM EDTA, pH 8.0, using *ε*_412_ = 14 150 M^−1^ cm^−1^ for free thiols.^66,87^ With the MT-3 protein concentration determined from the thiol content, the appropriate stoichiometry of Zn^2+^ from solutions of ZnCl_2_ was added to generate Zn_7_MT-3.

The individual α- and β-domains of MT-3 were purified in an identical manner. All constructs are listed in the ESI (Fig. S1†).

### Metallothionein-2 (MT-2) expression, purification and characterization

The recombinant human MT-2 expression plasmid was generated by synthetic DNA synthesis upon codon optimization (Genscript) and cloned in a pET-3d plasmid (Novagen) between the NcoI/BamHI restriction sites. MT-2 was expressed in *E. coli* BL21(DE3)pLys cells and purified following the method of Faller *et al.*^88^ In this method, MT-2 is expressed and purified in its Cd-bound form upon addition of 0.4 mM CdSO_4_ 30 min after IPTG induction. For the anion-exchange chromatography step, MT-2 was eluted with a linear gradient of 0 to 200 mM NaCl in 25 mM Tris, pH 8.6, at a flow-rate of 8 mL min^−1^ using a HiPrep DEAE FF 16/10 column connected to an Äkta Pure chromatographic system (GE Healthcare, now Cytiva). The Cd-containing fractions, as determined by ICP-MS quantification, were pooled. The apo-protein was then generated and reconstituted to the Zn_7_MT-2 form using the method of Vašák,^89^ by addition of HCl, followed by the addition of ZnCl_2_ and adjustment of the pH to 8.0 with 1 M Tris base. Zn_7_MT-2 was further purified by size exclusion chromatography to ensure the complete removal of low molecular weight protein contaminants. The MT-2 protein concentration was determined spectrophotometrically in 0.1 M HCl, which releases the Zn^2+^ to generate apo MT-2, using a Cary 300 UV-vis spectrophotometer (Agilent) and *ε*_220_ = 48 200 M^−1^ cm^−1^. The Zn^2+^ concentration was determined with quantification by ICP-MS (Agilent 7900), using protein samples digested in 45% HNO_3_ (v/v) overnight and subsequently diluted to 1% HNO_3_. The Cys concentration was determined spectrophotometrically by sulfhydryl group quantification, after sample reaction with 2,2-dithiodipyridine in 0.2 M sodium acetate, 1 mM EDTA, pH 4.0, using *ε*_343_ = 7600 M^−1^ cm^−1^ for free thiols.^87^ Zinc-to-protein ratios of 7.0 ± 0.5 and Cys-to-protein ratios of 20 ± 3 were obtained. The protein purity was confirmed by SDS-PAGE using MT samples subjected to cysteine modification by monobromobimane, following the method of Meloni *et al.*^62^

### ITC data collection and analysis

All ITC measurements were obtained with a Malvern (MicroCal) VP-ITC housed in a custom plexiglass glovebox under a N_2_ atmosphere at 25 ± 0.2 °C. The samples were stirred at a constant rate in the range 307 to 437 rpm and titrant injection volumes were 4 to 12 μL, with a spacing of 240 to 600 seconds between each injection. Heat associated with the last few injections quantifies the heat of dilution, which was subtracted from the heat of each injection. The ITC data are presented as the baseline adjusted heat flow *vs.* time in the upper panel and the integrated, concentration-normalized, molar heat per injection *vs.* molar ratio of titrant-to-titrand in the lower panel. Data were analysed by either a one-site or two-sites binding model with Origin 7.0 software. The tabulated experimental values represent the average and standard deviation from at least three independent measurements, unless otherwise noted. The change in entropy is reported as −*T*Δ*S* at 25 °C, which has the same units and sign convention as Δ*H*, for ready comparison of thermodynamic values.

Protein samples were prepared in buffer solutions that were identical to those containing the metal ion or chelate titrant. For Zn^2+^ chelation measurements, the chelate concentration was 120 to 140 times greater than the concentration of the Zn^2+^-bound protein sample (∼5 μM). For the Cu^+^ binding measurements, GSH was present at the same concentration in both titration solutions, with a 10 : 1 ratio of GSH to Cu^+^ (∼0.5 mM) in the syringe and a 2000 : 1 ratio of GSH to protein (∼2.5 μM) in the cell.

For each type of ITC measurement, the experimental change in enthalpy (Δ*H*_ITC_) in different buffers was used to quantify the number of protons that bind to, or are released from, the buffer upon addition of the titrant to the sample in the cell.^90^ This value was then used to determine the contribution of buffer protonation to Δ*H*_ITC_, so it could be subtracted. It was also used to quantify the number of protons displaced from the protein upon metal binding at the experimental pH. An analysis based on Hess's law was used to determine the metal-binding enthalpy from the Δ*H*_ITC_ value. The metal-binding equilibrium constant was determined from the experimental binding constant (*K*_ITC_) for direct metal titrations as described previously^48,91^ and for chelation titrations as described in the ESI.†

### Metal content and 77 K luminescence characterization of Cu_*n*_^+^MT-2 and Cu_*n*_^+^MT-3

The Cu_*n*_^+^MT species formed upon reaction of Cu^+^ with Zn_7_MT-2 or Zn_7_MT-3 in the presence of excess GSH were prepared to mimic the conditions utilized in the ITC measurements and correspond to the complete Cu^+^ titration of the MT samples. Zn_7_MT-2 and Zn_7_MT-3 stock solutions and the GSH-containing buffer (100 mM MOPS, pH 7.4, 150 mM NaCl, 20 mM GSH) were made oxygen-free by three vacuum/nitrogen cycles on a Schlenk line, while a stock solution of [Cu(MeCN)_4_]PF_6_ (100 mM in MeCN) was prepared inside a constant-flow nitrogen-purged anaerobic glovebox. A 10 mM Cu^+^ stock solution was subsequently prepared in the glovebox with the anaerobic GSH-containing buffer and used for reactions with the Zn_7_MT samples.

20 equivalents/mole of Cu^+^ were mixed with Zn_7_MT-2 (25 mM Tris, 50 mM NaCl, pH 8.0) and Zn_7_MT-3 (100 mM Bis–Tris, 150 mM NaCl, pH 7.4) samples (500 μL, 10 μM) and incubated for 2.5 h at 25 °C inside the nitrogen-purged glovebox. The mixtures were subsequently purified by injection onto a Superdex 75 10/300 column connected to an Äkta Pure Chromatographic System (GE Healthcare) and elution with 100 mM MOPS, pH 7.4, 150 mM NaCl, 20 mM GSH. The fractions corresponding to MT-2 or MT-3 were identified by subjecting aliquots from each peak of the SEC chromatogram to monobromobimane modification^62^ and SDS-PAGE to confirm the presence and purity of MT-2 or MT-3 (Fig. S2†).

The Cu- and Zn-to-MT stoichiometries of the purified MT samples were determined by metal and protein quantification. Cu and Zn were quantified by ICP-MS (Agilent 7900) after digesting the MT samples in 45% HNO_3_ (v/v) overnight at room temperature and subsequently diluting each sample to a final 1% HNO_3_ (v/v) with ultrapure H_2_O. Prior to determining the protein concentration, samples were acidified with 1 M HCl to pH < 1 to ensure metal release from the protein. The acidified samples were subsequently washed by three consecutive dilution–concentration cycles (3 × 500 μL) with 100 mM HCl in a 3 kDa cutoff Amicon Ultra centrifugal filter. Protein concentrations were determined spectrophotometrically on a Cary 300 UV-vis spectrophotometer (Agilent) by recording the absorbance at 220 nm and using *ε*_220_ = 53 000 M^−1^ cm^−1^ and 48 200 M^−1^ cm^−1^ for MT-3 and MT-2, respectively.

The formation and nature of the Cu^+^–thiolate clusters in the Cu_*n*_^+^MT samples were characterized by low temperature (77 K) luminescence emission spectra of the purified MT-2 and MT-3 fractions using a FluoroMax-4 spectrofluorometer (Horiba Scientific). 400 μL samples were flash frozen using liquid N_2_ inside a 4 mm inner diameter quartz tube, placed in a quartz dewar, and the emission spectra recorded from 380 to 750 nm (*λ*_exc_: 320 nm, slit width: 5 nm) with a 10 μs initial delay and a 300 μs sample window. The final spectra for each sample were recorded three times upon rotating the quartz tube inside the dewar and averaged to minimize freezing inhomogeneities. Spectra were subsequently collected for three independent sample replicates and the corresponding standard deviation calculated. Decay lifetimes of the emissive bands at 425 nm and 575 nm were determined on the same samples using a 75 μs initial delay and 300 μs sample window. Delay increments of 10 μs and 20 μs and maximum delays of 500 μs and 1000 μs were used for the 425 nm and 575 nm bands, respectively. Lifetimes at 425 nm and 575 nm for both MT-2 and MT-3 were calculated by fitting the data on three independent replicates with a single decay exponential function.

## Results, analysis, and discussion

Measurements of metal ions binding to metallothionein (MT) are challenging, due in large part to the susceptibility of the many conserved cysteine residues to oxidation. However, the Zn^2+^-bound form of the protein is less prone to oxidation and amenable to storage and reproducible characterization. Therefore, the thermodynamics of Zn^2+^ and Cu^+^ binding to MT-3 were determined with two types of anaerobic ITC measurements, both involving samples of Zn_7_MT-3. For Zn^2+^, the metal ion was extracted from the protein by titration with a chelating ligand whose affinity for Zn^2+^ is higher than that of the protein. This type of ITC measurement has been used previously to quantify the binding of metal ions to proteins,^91^ including MT-3.^48^ The thermodynamics of Zn^2+^ binding to MT-3 were then determined from an analysis of the chelation experimental data that includes competition between the chelating ligand and MT-3 for the Zn^2+^ and the concept of microscopic reversibility. Since MT has a higher affinity for Cu^+^ than it does for Zn^2+^, titration of the former into samples of Zn_7_MT-3 results in displacement of the latter.^4,36^ However, Cu^+^ is unstable to disproportionation in aqueous solution, which can be suppressed by the addition of a ligand that is introduced for this purpose.^92^ The thermodynamics of Cu^+^ binding to MT-3 were determined with an analysis that accounts for competition between the Cu^+^-stabilizing ligand and MT-3 for Cu^+^, as well as Zn^2+^ in the initial Zn_7_MT-3 sample. The resulting Cu^+^-bound protein was characterized further by ICP-MS to determine the Cu-to-protein stoichiometry and by low temperature luminescence to provide information on the structure of copper clusters in the protein that results under these conditions.

### Zn^2+^ binding to MT-3

Our previous ITC measurements of the thermodynamics of Zn^2+^ binding to MT-3 were part of a study of Pb^2+^ binding to the protein, which was conducted at pH 6.0 to ensure the Pb^2+^ remained soluble.^48^ In that study, the challenges of working with metal-free (apo) MT-3 were avoided by titrating the chelating ligand ethylenediaminetetraacetic acid (EDTA) into Zn_7_MT-3 and analysing the experimental binding isotherms with microscopic reversibility. The data revealed three sequential chelation events with different enthalpies, which were analysed to quantify the thermodynamics of triphasic Zn^2+^ binding to apo MT-3 to form Zn_7_MT-3 at pH 6.0. For the current study, similar measurements with EDTA at pH 7.4 gave different results that were not readily interpreted, revealing complex and pH-dependent equilibria for chelation titrations with EDTA. Therefore, other chelating ligands were evaluated for ITC measurements at pH 7.4, and diethylenetriaminepentaacetic acid (DTPA), which binds Zn^2+^ with an affinity that is two orders of magnitude higher than that of EDTA, was found to give consistent results.

**Fig. 2:**
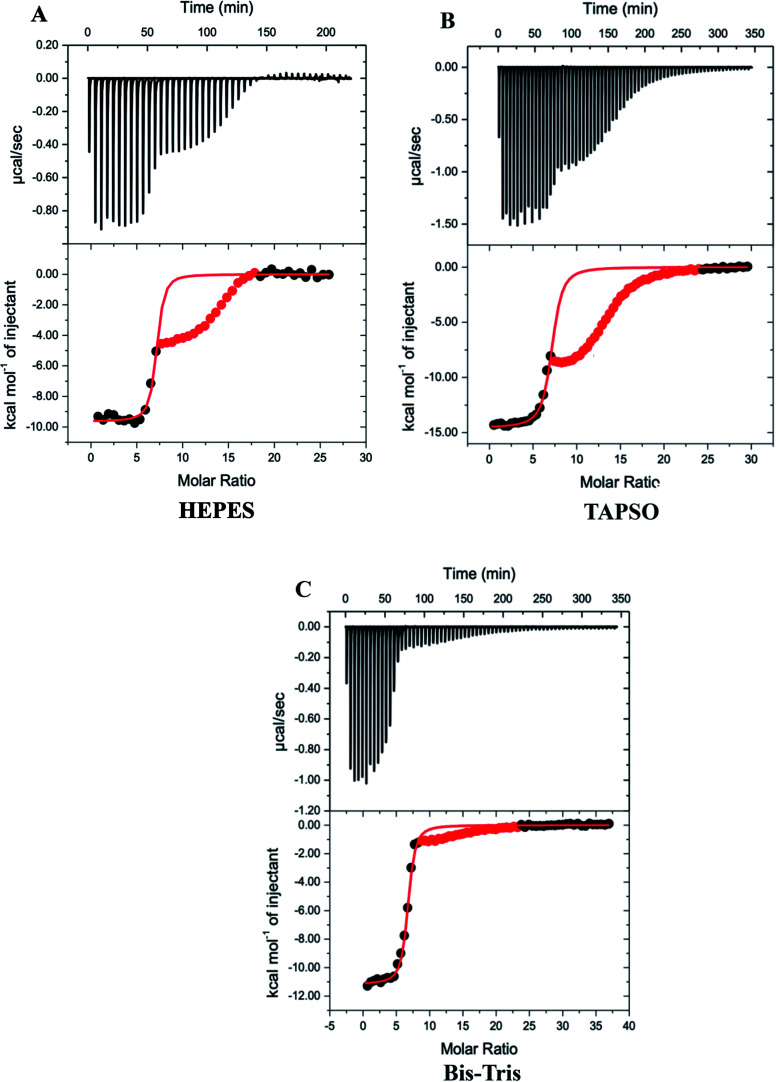
Representative thermograms for DTPA chelation of Zn^2+^ from Zn_7_MT-3 in 100 mM buffer and 150 mM NaCl at pH 7.4; data for the second event are masked (red) and data for the first event were fitted (solid line) to a one-site binding mode with the best-fit values and fit errors: (A) HEPES: *n*_ITC_ = 6.93 ± 0.05, *K*_ITC_ = 1.2 (±0.3) × 10^7^ and Δ*H*°_ITC_ = −9.64 ± 0.09 kcal mol^−1^; (B) Bis–Tris: *n*_ITC_ = 6.56 ± 0.01, *K*_ITC_ = 5.6 ± 0.3 × 10^6^ and Δ*H*°_ITC_ = −11.16 ± 0.04 kcal mol^−1^; (C) TAPSO: *n*_ITC_ = 7.02 ± 0.04, *K*_ITC_ = 3.9 (±0.5) × 10^6^ and Δ*H*°_ITC_ = −14.55 ± 0.07 kcal mol^−1^.

The DTPA titrations of Zn_7_MT-3, however, are unusual in that they have both a net exothermic chelation of Zn^2+^ with an inflection at the stoichiometry of the Zn^2+^ in the Zn_7_MT-3 sample, followed by another exothermic event upon further addition of DTPA, suggesting the formation of a higher-order DTPA–Zn^2+^ complex (Fig. 2). While initially puzzling, control titrations of DTPA into Zn^2+^ in the same buffers at pH 7.4 revealed a similar pattern, with initial formation of the 1 : 1 DTPA–Zn^2+^ complex and subsequent formation of a 3 : 2 DTPA–Zn^2+^ complex, whose formation enthalpy depends on the buffer that is present (Fig. S3†). This latter observation provides an important correlation to the MT-3 data, as the magnitude of the formation enthalpy for this subsequent DTPA–Zn^2+^ species in different buffers is Bis–Tris < HEPES < TAPSO < Tris for both the control titrations and the protein titrations. Therefore, the isotherms for DTPA titrations of Zn_7_MT-3 also contain the subsequent formation of a species when excess DTPA is added to the DTPA–Zn^2+^ formed by chelation of Zn^2+^ from the protein.

Since the first event in the experimental titrations is the chelation of Zn^2+^ from Zn_7_MT-3, data after the inflection were masked (red points) and only data associated with the initial inflection were fitted to a one-site binding model (Fig. 2). In contrast to ITC data at pH 6.0, where EDTA chelation isotherms contain three inflections and were fitted with a three-site binding model,^48^ the DTPA chelation of each Zn^2+^ at pH 7.4 has the same net change in enthalpy as each metal ion is removed from the protein at this pH under these conditions. The average best-fit experimental values for titrations in three different buffers are found in Table 1.

**Table 1:** Average best-fit experimental values for DTPA chelation of Zn^2+^ from Zn_7_MT-3, Zn_4_αMT3 and Zn_3_βMT-3 in the indicated buffers, obtained from fits of the first binding event, which is chelation of Zn^2+^ from the protein

Protein	Buffer	*n*_ITC_	*K*_ITC_	Δ*H*°_ITC_ (kcal mol^−1^)
Zn_7_MT-3	HEPES	6.90 ± 0.05	1.27 (±0.09) × 10^7^	−9.5 ± 0.2
	Bis–Tris	6.5 ± 0.1	1.1 (±0.9) × 10^7^	−11.0 ± 0.2
	TAPSO	7.08 ± 0.06	4.5 (±0.6) × 10^6^	−14.1 ± 0.7

Zn_4_αMT-3	Bis–Tris	4.4 ± 0.3	9 (±5) × 10^7^	−10.1 ± 0.6
	TAPSO	4.2 ± 0.2	6.5 (±0.3) × 10^6^	−14.1 ± 0.1

Zn_3_βMT-3	Bis–Tris	2.6 ± 0.1	2.8 (±0.6) × 10^7^	−10.9 ± 0.5
	TAPSO	2.8 ± 0.2	4 (±1) × 10^6^	−14.1 ± 0.4

The experimental ITC values obtained from chelation titrations require additional analysis to determine the buffer- and chelate-independent thermodynamics of Zn^2+^ binding to MT-3 at pH 7.4. This *post hoc* analysis takes into account the contributions from coupled and competing equilibria. For the enthalpy, these include deprotonation of the DTPA chelating ligand (Δ*H*_LH1_ and Δ*H*_LH2_), protonation of the buffer (Δ*H*_BH_), the desired dissociation of Zn^2+^ from MT-3 (Δ*H*_MP_), protonation of MT-3 upon release of Zn^2+^ (Δ*H*_PH_) and Zn^2+^ binding to DTPA (Δ*H*_ML_). This analysis requires the net protonation, or deprotonation, of the buffer, which is determined from the dependence of the experimental enthalpy (Δ*H*_ITC_) on the buffer protonation enthalpy (Δ*H*_BH_) for data in different buffers (Fig. S4†). This value, which is determined from the slope of these plots, allows the enthalpic contribution from buffer protonation to be subtracted and, through a proton inventory (Scheme S1†), the protonation of MT-3 upon release of Zn^2+^ to be quantified (9.5 ± 0.3 H^+^). As summarized in Scheme S1,† the weighted sum of the enthalpies for each of these events equals the experimentally measured enthalpy (Δ*H*_ITC_). The condition-independent Zn^2+^-binding enthalpy determined here is the average value per Zn^2+^ for the formation of Zn_7_MT-3 at pH 7.4: 7Zn^2+^ + MT-3 → Zn_7_MT-3 + 9.5H.

To determine the buffer- and chelate-independent binding constant for Zn^2+^ binding to MT-3, coupled and competing equilibria must be taken into account in a *post hoc* analysis similar to that for the binding enthalpy, as outlined in the ESI.† The results of these analyses of the net experimental binding enthalpy (exothermic) and binding constant to determine the condition-independent binding enthalpy (endothermic) and binding constant provide the thermodynamic values for Zn^2+^ binding to MT-3 at pH 7.4 found in Table 2.

**Table 2:** Buffer-independent thermodynamics of Zn^2+^ binding to MT-3 and to the individual α and β domains at pH 7.4 and 25 °C

Protein	*n* (Zn^2+^)	*K*	Δ*G*° (kcal mol^−1^ Zn^2+^)	Δ*H*° (kcal mol^−1^ Zn^2+^)	−*T*Δ*S*° (kcal mol^−1^ Zn^2+^)
MT-3	6.8 ± 0.3	4 (±2) × 10^11^	−15.7 ± 0.3	13.4 ± 0.2	−29 ± 0.5
αMT-3	4.3 ± 0.3	4 (±3.5) × 10^11^	−15 ± 1	4.26 ± 0.05	−20 ± 1
βMT-3	2.7 ± 0.2	9 (±8.5) × 10^11^	−16.0 ± 0.8	5.76 ± 0.06	−21.8 ± 0.8

Data from our earlier study of Zn^2+^-binding to MT-3 at pH 6.0 revealed that Zn^2+^ binds to MT-3 sequentially in three populations with different enthalpies, an initial ∼4 that bind most tightly with terminal tetra-thiolate coordination, a subsequent ∼2 that bind less tightly with bridging thiolate coordination and finally ∼1 that binds even less tightly.^48^ For DTPA chelation measurements at pH 7.4, we measure only a single set of thermodynamic values for all seven Zn^2+^ ions bound to MT-3. This result is clearly different from our chelation ITC measurements at lower pH, as well as results from chromophore chelation measurements and fitting in an earlier study of MT-2 by Krężel and Maret.^93^ While the DTPA chelation data could suggest a cooperative chelation process at pH 7.4, this would be inconsistent with other experimental results (*e.g.* zinc titrations followed by ESI-MS analysis) and computational simulations of MTs binding and releasing Zn^2+^.^93–95^ Of relevance is the Zn^2+^ affinity of the chelate used. The chromophore chelates have affinities that are lower than that of MT, while DTPA has an affinity that is 6–7 orders of magnitude greater than that of MT, as required for ITC measurements, and two orders of magnitude greater than that of EDTA. The much higher Zn^2+^ affinity of DTPA and the properties of Zn_7_MT-3 at pH 7.4 result in conditions where the ITC chelation measurements are only able to discern an average Zn^2+^ affinity of MT-3. This value, which is obtained from the *post hoc* analysis of the ITC chelation data (*K* = 4 (±2) × 10^11^ at pH 7.4), is comparable to two reported values (*K* = 6.2 × 10^10^ at pH 8.0 and *K* = 2.4 × 10^11^ for the β-domain)^96,97^ but somewhat higher than another (*K* = 7.7 × 10^9^).^57^

The binding of Zn^2+^ to MT-3 at pH 7.4 is enthalpically disfavoured (Δ*H* = +13 kcal mol^−1^) and entropically driven (Δ*S* = 97 cal mol^−1^ K^−1^; −*T*Δ*S* = −29 kcal mol^−1^ at 25 °C). This is similar to results for Zn^2+^ binding to peptides with tetra-thiolate ligation, including a Gly-rich peptide prepared and studied by Gibney and co-workers^98^ (Δ*H* = +5.6 kcal mol^−1^; Δ*S* = 77 cal mol^−1^ K^−1^; −*T*Δ*S* = −23 kcal mol^−1^ at 25 °C) and a peptide corresponding to the C-terminal Zn-binding sequence of the glucocorticoid receptor (GR-2) studied by Rich *et al.*^99^ (Δ*H* = +10 ± 2 kcal mol^−1^; Δ*S* = 70 cal mol^−1^ K^−1^; −*T*Δ*S* = −20 ± 2 kcal mol^−1^ at 25 °C). The net endothermic binding is attributed to a large contribution from the enthalpic penalty to deprotonate the Cys thiols. The net increase in entropy is attributed to desolvation of the Zn^2+^ ions and protein and loss of the Cys protons, in spite of conformational restrictions imposed on the protein by Zn^2+^ coordination.

Further analysis of the Zn^2+^-binding thermodynamics of MT-3, however, reveals an important, and previously unknown, property of this protein. The endothermic contribution from deprotonation of the Cys thiols can be subtracted from the net Zn^2+^ binding enthalpy to estimate the Zn^2+^–thiolate bond enthalpy. When this penalty is subtracted from data for the tetra-Cys peptide GR-2,^99^ the residual enthalpy is −25 kcal mol^−1^ Zn^2+^. When a similar subtraction is made for Cys thiols that are deprotonated upon Zn^2+^ binding to MT-3, the corresponding value is +1.5 kcal mol^−1^ Zn^2+^ (Δ*H*_MP_ in Scheme S1†). The enthalpy of a μ_2_-bridging Zn^2+^–thiolate bond, which is the case for some of the Zn^2+^ in MT, is expected to be smaller than that of a terminal Zn^2+^–thiolate bond and thus the average bond enthalpy for Zn^2+^ ions bound to MT-3 should be less than that of tetra-thiolate coordination. However, the enthalpy of Zn^2+^–thiolate bonds must be favourable (negative) for stable binding. Therefore, this unusual result for MT-3 reveals there must be another endothermic contribution to Zn^2+^ binding to MT-3 (*vide infra*).

The high affinity of MT-3 for Zn^2+^ is due to a very favourable change in entropy upon binding the metal ion, which is even more favourable than that of Zn^2+^ binding to tetra-Cys peptides. The two cases would have similar entropic contributions per Zn^2+^ from desolvation of the metal ion and an inventory of species reveals a comparable net increase in the translational entropy (eqn (1) and (2)),

1 MT-3 + 6.8 Zn^2+^ → Zn_6.8_MT-3 + 9.5 H^+^, Δ_species_ = 10.5 − 7.8 = +2.7

2 GR-2 + Zn^2+^ → ZnGR-2 + 4H^+^, Δ_species_ = 5 − 2 = +3

although this contribution may be small.^100,101^ A major difference between MT-3 and the tetra-Cys peptides, however, is the penalty for conformational restrictions upon Zn^2+^ binding, which is high for unstructured peptides. The −*T*Δ*S* value for MT-3 is an average per metal for formation of Zn_7_MT-3. A significant loss of protein conformational entropy is expected initially as the first Zn^2+^ ions bind, but a smaller protein penalty is expected as subsequent Zn^2+^ bind with bridging coordination to form the Zn_3_ and Zn_4_ clusters in the β and α domains, respectively. Thus, a lower protein conformational penalty per Zn^2+^ is expected to contribute to the net change in entropy for Zn_7_MT-3 formation.

### Zn^2+^ binding to the individual domains of MT-3

**Fig. 3:**
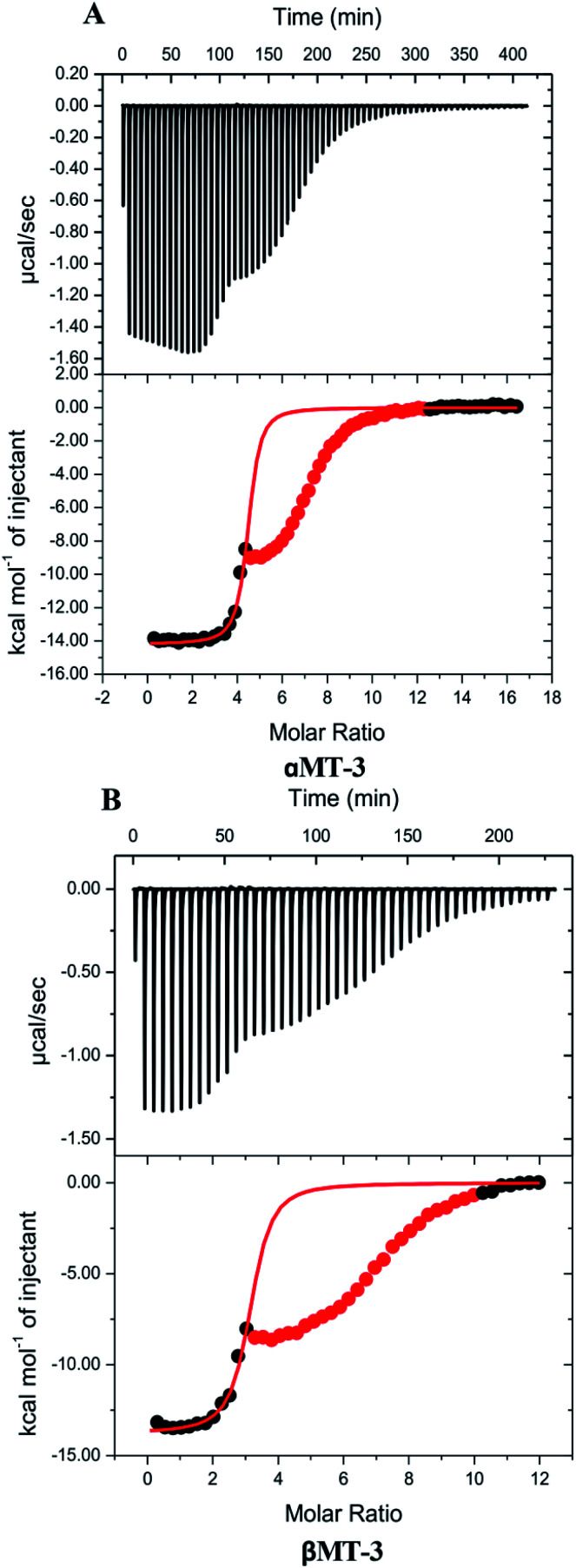
Representative thermograms of the DTPA chelation of Zn^2+^ in 100 mM TAPSO buffer and 150 mM NaCl at pH 7.4; data for the second event are masked (red) and data for the first event were fitted (solid line) to a one-site binding model with best-fit values and fit errors: (A) Zn_4_αMT-3: *n*_ITC_ = 4.39 ± 0.02, *K*_ITC_ = 6.7 (±0.9) × 10^6^ and Δ*H*°_ITC_ = −14.19 ± 0.06 kcal mol^−1^; (B) Zn_3_βMT-3: *n*_ITC_ = 3.07 ± 0.04, *K*_ITC_ = 4.1 (±0.9) × 10^6^ and Δ*H*°_ITC_ = −13.8 ± 0.1 kcal mol^−1^.

Quantitative insight on inter-domain interactions of MT-3 can be obtained from measurements of metal ions binding to the individual domains for comparison to results on MT-3. Fig. 3 shows ITC data for DTPA titrations of Zn_4_αMT-3 and Zn_3_βMT-3 at pH 7.4. Similar to the whole protein, isotherms of the individual domains have an initial exothermic event with a stoichiometry that matches the Zn^2+^ bound to the domain in the sample, followed by the exothermic binding of excess DTPA to the DTPA–Zn^2+^ from the Zn^2+^ chelation. As with Zn_7_MT-3, only the data associated with the first event were fitted with a one-site binding model. Average best-fit experimental ITC values for DTPA chelation of Zn^2+^ from Zn_4_αMT-3 and Zn_3_βMT-3 in two different buffers are found in Tables S1 and S2,† respectively.

Our ITC data for Zn^2+^ binding to the individual α and β domains of MT-3 allow us to evaluate the thermodynamics of inter-domain interaction.^95,102,103^ The results in Table 3 show that the free energy of Zn_7_MT-3 formation is identical to the sum of the free energies of formation of Zn_4_αMT-3 and Zn_3_βMT-3. However, there are significant enthalpic and entropic differences between MT-3 and the sum of the values for the isolated domains, with an unfavourable difference in the formation enthalpy (ΔΔ*H* = +57 kcal mol^−1^ protein) and a favourable difference in the formation entropy (−*T*ΔΔ*S* = −52 kcal mol^−1^ protein at 25 °C) when the two domains are linked in the protein. This reveals that domain–domain interactions provide an entropic benefit but an enthalpic penalty to Zn^2+^ binding to form the Zn_4_ cluster in the α-domain and the Zn_3_ cluster in the β-domain. These two effects, however, are similar in magnitude, so they cancel, in another example of enthalpy–entropy compensation (EEC) in biological coordination chemistry.^98,99^

**Table 3:** Thermodynamic values for the formation of Zn_7_MT-3, Zn_4_αMT-3 and Zn_3_βMT-3 at pH 7.4 and 25 °C, and the sum of the values for the two domains and the difference between the MT-3 values and the sum of the domain values

Sample	*n* (Zn^2+^)	*n* (H^+^)	Δ*G*° (kcal mol^−1^)	Δ*H*° (kcal mol^−1^)	−*T*Δ*S*° (kcal mol^−1^)
MT-3	6.8 ± 0.3	9.5 ± 0.3	−106.8	91.1	−197.2
α domain	4.3 ± 0.3	7.0 ± 0.6	−64.5	18.3	−86.0
β domain	2.7 ± 0.2	4.1 ± 0.4	−43.2	15.6	−58.9
*α* value + *β* value	7.0 ± 0.4	11.1 ± 0.7	−107.7	33.9	−144.9
Difference: MT-3 − (*α* + *β*)	—	—	+0.9	+57.2	−52.3

In the analysis above of the net enthalpy of Zn^2+^ binding to holo MT-3, subtraction of the endothermic Cys deprotonation gave an enthalpy, which is expected to be that of Zn^2+^ binding to the Cys thiolates, that was unexpectedly endothermic (+1.5 kcal mol^−1^ Zn^2+^). A similar analysis that subtracts the Cys thiol deprotonation enthalpy from the net Zn^2+^-binding enthalpy of the isolated α and β domains gives the favourable values of −9.6 and −7.1 kcal mol^−1^ Zn^2+^, respectively. This supports the notion that the endothermic (unfavourable) value for the whole MT-3 contains an additional endothermic contribution, which appears to be the endothermic inter-domain interaction (+57 kcal mol^−1^ protein). In fact, when this value is put on a per Zn^2+^ basis (+8.4 kcal mol^−1^ Zn^2+^) and subtracted from the Zn^2+^–thiolate binding enthalpy, a value of −6.9 kcal mol^−1^ Zn^2+^ is now found for MT-3. Thus, while the thermodynamics of interdomain interaction do not impact the affinity of MT-3 for Zn^2+^, they do modulate the relative contributions of the binding enthalpy (disfavouring) and binding entropy (favouring) to the affinity of this two-domain protein for metal ions.

### Cu^+^ binding to MT-3 and MT-2

Quantitative measurements of Cu^+^ coordination chemistry in aqueous solution are challenging due to its ready oxidation to Cu^2+^ and favourable disproportionation to Cu^2+^ and Cu^0^. While the former can be prevented with anaerobic conditions, the latter needs to be suppressed by a ligand that favours Cu^+^ over Cu^2+^, such as 1,1,4,7,10,10-hexamethyltriethylenetetramine (Me_6_Trien), bicinchoninic acid (BCA), bathocuproine disulfonate (BCS) or glutathione (GSH).^92^ Since the Cu^+^-stabilizing ligand competes with the protein for Cu^+^, it was necessary to choose a ligand with an affinity for Cu^+^ that is less than that of the protein, yet one whose competition with MT-3 falls within the accuracy range of the titration calorimeter (∼3 < log *K*_competition_ < ∼8). After initial measurements in MeCN (see ESI†), GSH proved to be ideal and Cu^+^ binding to MT-3 was measured with Cu^+^ titrations of Zn_7_MT-3 in a solution containing excess GSH, the only physiologically relevant ligand from among those noted above. To minimize the heat of dilution in the ITC measurements, the concentration of GSH was matched in the titrant and titrand solutions. Therefore, the 10-fold excess of GSH to Cu^+^ in the syringe required a ∼2000-fold excess of GSH to Zn_7_MT-3 in the cell. Based on the affinity of MT-3 for Zn^2+^ determined here (log *K* = 11.6) and the stability of Zn^2+^(GSH)_2_ (log *β*_2_ ∼ 12),^104^ we estimate pZn^2+^_free_ = 8.2 for the former equilibrium and pZn^2+^_free_ = 10.6 for the latter equilibrium under our experimental conditions. These values indicated that the large excess of GSH would remove Zn^2+^ from Zn_7_MT-3 *in situ*. The resulting apo MT-3 appears to be stable in the excess GSH for the duration of the anaerobic ITC measurement, as there was no evidence of protein precipitation in the samples or the ITC isotherms (excessive heats, baseline noise, *etc.*) and the results were reproducible.

**Fig. 4:**
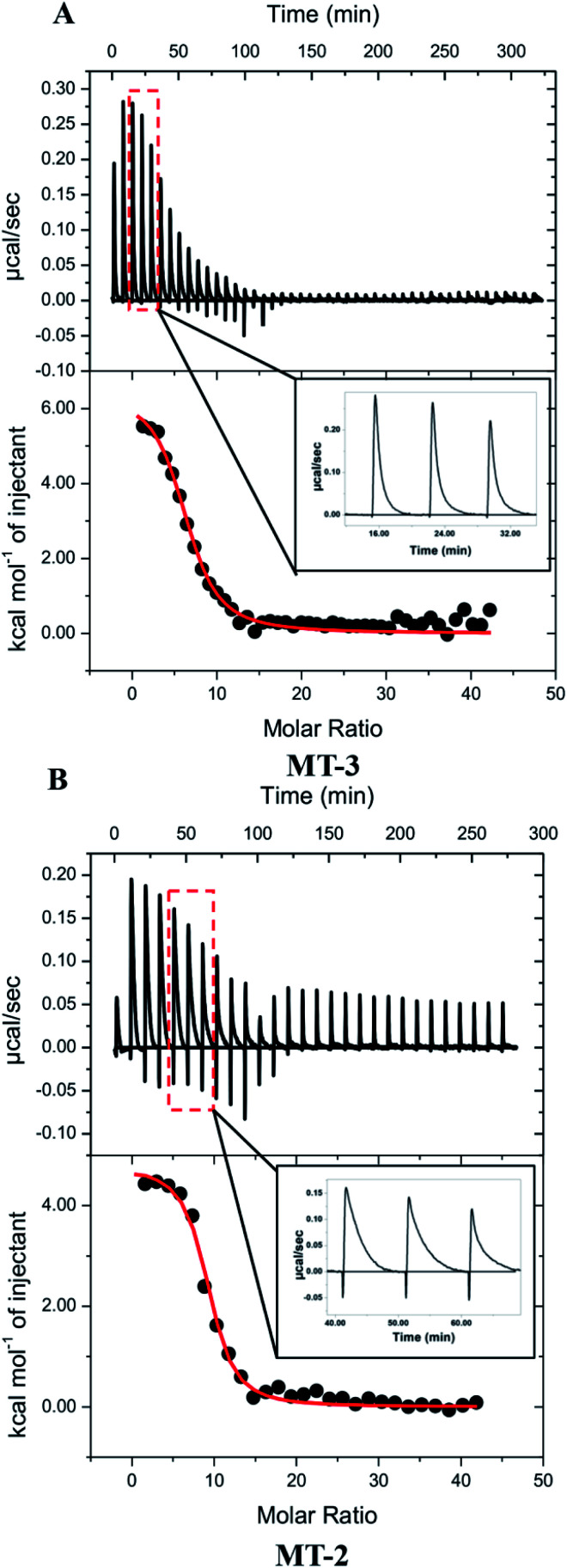
Representative thermograms of Cu^+^ titrated into (A) Zn_7_MT-3 and (B) Zn_7_MT-2 in 100 mM buffer, 150 mM NaCl and 5 mM GSH at pH 7.4; the data were fitted (solid line) to a one-site binding model with the best-fit values and fit errors for Zn_7_MT-3 in Bis–Tris buffer: *n*_ITC_ = 6.5 ± 0.2, *K*_ITC_ = 6 (±1) × 10^5^ and Δ*H*°_ITC_ = 6.5 ± 0.3 kcal mol^−1^, and for Zn_7_MT-2 in MOPS buffer are *n*_ITC_ = 8.9 ± 0.1, *K*_ITC_ = 1.4 (±0.2) × 10^6^ and Δ*H*°_ITC_ = 4.8 ± 0.1 kcal mol^−1^; insets show enlargements of representative injection peaks, which plot μcal s^−1^*vs.* time.

Representative ITC data for Cu^+^ titrated into Zn_7_MT-3 with both titrant and titrand in a buffered 5 mM GSH solution are shown in Fig. 4A, where the binding isotherm reflects the competition between the protein and GSH for Cu^+^. The isotherms were fit to a one-site binding model, indicating similar thermodynamics for all Cu^+^ binding to MT-3 under these conditions, with average experimental ITC values from data in three buffers found in Table 4. Further analysis of these net endothermic data that accounts for coupled and competing equilibria requires the quantification of buffer protonation upon Cu^+^ binding. The experimental binding enthalpy in solutions with different buffers was used in this analysis (Fig. S5A†), which shows that 0.5 ± 0.2 protons dissociate from the buffer upon Cu^+^ binding to the protein at pH 7.4. Since the two GSH that are initially bound to each Cu^+^ would bind 1.6 ± 0.2 protons after the Cu^+^ has been released at this pH, a proton inventory (Scheme S2†) shows that 9.1 ± 0.2 protons are displaced from MT-3 when 6 ± 1 Cu^+^ ions bind to the protein at pH 7.4 in these ITC measurements. If Zn^2+^ had remained bound to MT-3 in the presence of the large excess GSH, then Cu^+^ would have displaced the Zn^2+^ without the release of protons. This confirms that Cu^+^ is, in fact, binding to *in situ* generated apo MT-3 under these conditions.

**Table 4:** Average best-fit experimental values for the addition of Cu^+^ in excess GSH to Zn_7_MT-3 and Zn_7_MT-2 in the indicated buffers, obtained from the fits of data from at least two independent measurements to a one-site binding model

Protein	Buffer	*n*_ITC_	*K*_ITC_	Δ*H*°_ITC_ (kcal mol^−1^)
MT-3	MOPS	6.3 ± 0.7	5 (±1) × 10^7^	4.5 ± 0.7
	Bis–Tris	6 ± 1	4 (±2) × 10^5^	6.6 ± 1.5
	TAPSO	4.7 ± 0.2	2.1 (±0.2) × 10^6^	6.9 ± 0.5

MT-2	MOPS	8.5 ± 1.5	6 (±1) × 10^5^	3.6 ± 0.2
	TAPSO	8.1 ± 0.9	1.5 (±0.4) × 10^6^	5.2 ± 0.4

The condition-independent thermodynamic values for Cu^+^ binding to MT-3 were obtained from a *post hoc* analysis, which includes the competition between GSH and MT-3 for Cu^+^ and the protonation of GSH when Cu^+^ binds to the protein (Scheme S2†). These condition-independent values for Cu^+^ binding to MT-3 at pH 7.4 are found in Table 5. The average Cu^+^ affinity of MT-3 (log *K* = 19.6) is comparable to that of several Cu^+^-binding proteins with thiolate coordination^105–107^ and very similar to log *K* = 19.3 reported by Calvo *et al.*^77^ for MT-3 determined with a different method. The binding of Cu^+^ is both enthalpically favoured (exothermic) and entropically favoured (Table 5). Further analysis of the Cu^+^ binding enthalpy that subtracts the endothermic penalty for Cys deprotonation reveals an average enthalpy for binding to Cys thiolates of −23 kcal mol^−1^ Cu^+^, which gives an estimate of −7 kcal mol^−1^ for the average Cu^+^–thiolate bond enthalpy. The average Cu^+^ binding entropy is also favourable, although not as much so as the Zn^2+^ binding entropy. This is due, in part, to the smaller desolvation contribution from a monopositive, instead of a dipositive, metal ion binding to the protein. The contribution from translational entropy is comparable to that for Zn^2+^ binding (eqn (1)*vs.*(3)).

3 MT-3 + 6 Cu^+^ → Cu_6_MT-3 + 9.1 H^+^ Δ_species_ = 10.1 − 7 = +3.1

**Table 5:** Buffer-independent thermodynamics of Cu^+^ binding to MT-3 and MT-2 at pH 7.4 and 25 °C

Protein	*K*	Δ*G*° (kcal mol^−1^ Cu^+^)	Δ*H*° (kcal mol^−1^ Cu^+^)	−*T*Δ*S*° (kcal mol^−1^ Cu^+^)
MT-3	4 (±4) × 10^19^	−26.9 ± 0.5	−10 ± 1	−17 ± 1.5
MT-2	8 (±5) ± 10^19^	−27.1 ± 0.4	−12 ± 1	−15 ± 1

Calorimetric data for Cu^+^ titrations into Zn_7_MT-2 were obtained with conditions identical to those used with Zn_7_MT-3 (Fig. 4B and Table 4). An analysis of buffer protonation for the Cu^+^ titrations (Fig. S5B†) and a proton inventory showed that 12.4 ± 0.2 H^+^ are displaced from MT-2 when 8 ± 1 Cu^+^ ions bind to the protein at pH 7.4 in these ITC measurements. Therefore, as was the case with MT-3, the large excess of GSH extracts Zn^2+^ from Zn_7_MT-2 and these data quantify Cu^+^ binding to *in situ* generated apo MT-2. The condition-independent thermodynamics of Cu^+^ binding to MT-2 at pH 7.4 were then determined from a *post hoc* analysis of the binding enthalpy and binding constant, similar to that used for MT-3 (Table 5).

To the extent that ITC measurements of Cu^+^ binding can distinguish thermodynamic differences, Cu^+^ coordination by MT-3 and MT-2 are not significantly different in the presence of excess GSH, affirming that metal coordination is predominantly dictated by the conserved Cys residues in all mammalian MT isoforms. Nevertheless, a clear difference between the binding of Cu^+^ to MT-3 and MT-2 is seen in the experimental ITC data. In contrast to the monophasic injections with MT-3, the MT-2 measurements show biphasic injections, which are attributed to a rapid exothermic contact binding followed by a slower endothermic (protein) reorganization to the final equilibrium (Fig. 4, insets). This difference between MT-2 and MT-3 reflects different kinetics of Cu^+^ binding under these conditions and is consistent with other reports of unique metal-binding kinetics^77^ and metalloprotein dynamics^96^ for MT-3.

The reported Cu^+^-binding stoichiometries of MTs range from 4 to 20, with the maximum corresponding to a 1 : 1 Cu^+^ : Cys ratio, depending on the conditions and the type of measurement.^108–112^ The presence of excess GSH in our measurements limits the stoichiometry of bound Cu^+^ to those whose coordination to the protein is more stable than that of the Cu^+^ (GSH)_2_ complex (log *β*_2_ = 15.3),^104^ a point we believe is relevant to *in vivo* conditions.^113,114^ While the average Cu^+^ affinity and binding thermodynamics of MT-3 and MT-2 are comparable, within experimental error (Table 5), the number of Cu^+^ that bind to MT-3 (6 ± 1) and MT-2 (8 ± 1) that were determined by ITC measurements are different, as are the number of protons that are displaced upon formation of Cu_6±1_MT-3 (9.1 ± 0.2) and Cu_8±1_MT-2 (12.4 ± 0.2).

Binding stoichiometries determined from ITC measurements are affected by conditions (rapid stirring) and several sources of error (concentrations, volumes) and can have low accuracy and precision. Therefore, to accurately determine the stoichiometry of the resulting Cu^+^-bound MT-3 and MT-2, ICP-MS was employed. For both MT-3 and MT-2, quantification of the copper and zinc content by ICP-MS and analysis of the protein concentration allowed the Cu- and Zn-to-protein ratios in the final reaction products to be determined. These results show that the Cu : MT ratios were 8 : 1 mol mol^−1^ for both isoforms (8.5 ± 0.2 mol mol^−1^ for MT-3; 7.8 ± 0.2 mol mol^−1^ for MT-2), confirming the presence of Cu_8_^+^MT and suggesting the presence of two Cu_4_^+^ clusters, one in each domain. The somewhat lower Cu^+^ stoichiometry of MT-3 that was determined with ITC measurements is likely due to a small amount of protein oxidation. This would lower both the number of Cu^+^ that bind and the H^+^ that are displaced but not significantly affect the Cu^+^ affinity and binding thermodynamics, which are determined on a per Cu^+^ basis. Finally, zinc was not detected in the reaction products (<0.1 mol mol^−1^ for both MT-3 and MT-2), in agreement with Cu^+^ binding to apo MTs under the conditions used for ITC measurements.

**Fig. 5:**
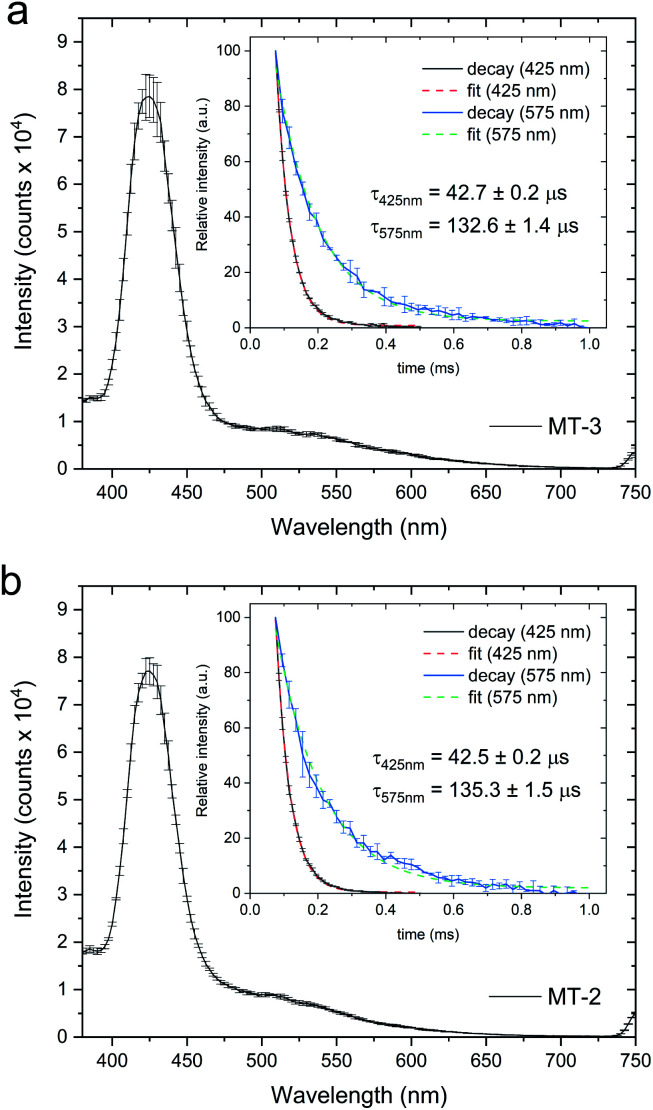
77 K luminescence emission spectra of the MT-3 (a) and MT-2 (b) fractions generated upon incubation of Zn_7_MT-3 or Zn_7_MT-2 (10 μM) with Cu^+^ (200 μM) in the presence of excess GSH (20 mM), followed by MT separation by SEC; insets are the emission decay and lifetimes (*τ*) at 425 nm and 575 nm obtained by fitting the data with a single decay exponential function.

Cu_4_^+^–thiolate clusters are readily identified by intense and characteristic low temperature luminescence spectra and emission lifetimes. For Cu_4_^+^-cluster(s) in MTs, this includes a sharp emission band at 425 nm with a triplet cluster-centered (CC) origin and a broad emission band at 560–575 nm with a triplet charge-transfer (CT) origin. The presence of the triplet CC band at 425 nm, with a characteristic lifetime, is indicative of short Cu^+^–Cu^+^ distances (<2.8 Å), d^10–^d^10^ overlap and metal–metal bonding, and is absent in clusters with higher nuclearity (*i.e.*, Cu_6_^+^–thiolate clusters in MTs).^82,115–117^ Low temperature (77 K) luminescence measurements for both MT-3 and MT-2 samples prepared by Cu^+^ addition to Zn_7_MT in excess GSH show emission features at 425 and 575 nm (Fig. 5), with the emission peak at 425 nm diagnostic of Cu_4_^+^–thiolate cluster formation.^82,83^ The lifetimes of these emission bands in both protein samples (Fig. 5, inserts) are also consistent with Cu_4_^+^ clusters. Thus, Cu^+^ titrations of Zn_7_MT-3 and Zn_7_MT-2 in the presence of excess GSH result in the formation of β(Cu^+^)_4_α(Cu^+^)_4_MT-3 and β(Cu^+^)_4_α(Cu^+^)_4_MT-2.

## Conclusions

### Biological implications from Zn^2+^ and Cu^+^ binding thermodynamics

A goal of *in vitro* measurements of protein coordination chemistry is to provide molecular insight on the *in vivo* binding of metals. A putative biological role for MT-3 is scavenging copper, which could be Cu^+^ in neuronal cells or Cu^2+^ from intracellular proteins under conditions of excess copper or from extracellular neuronal tissue.^61,80,81,118,119^ The thermodynamics of Cu^2+^ scavenging are complicated by reduction of the Cu^2+^ by Cys residues of MT-3, or other thiol-containing species,^70^ prior to Cu^+^ binding to the protein, which then contains oxidized Cys disulfide bonds. Calorimetric measurements for this proposed binding are difficult to interpret as they involve both redox chemistry and metal ion binding. Efforts to determine the formation thermodynamics of the native Cu_4_Zn_3–4_MT-3 through selective chelation of Zn^2+^ with EDTA and Cu^+^ with BCS in ITC measurements have so far proven to be challenging. Nevertheless, by individually quantifying the thermodynamics of Cu^+^ and Zn^2+^ binding to MT-3, we can determine the competition between these two metal ions for the protein. Table 6 compares the Cu^+^ and Zn^2+^ binding thermodynamics at pH 7.4, thereby quantifying Cu^+^ displacement of Zn^2+^ as the difference between their MT-3 binding thermodynamics. This reveals that the entropically favoured binding of Zn^2+^ (−*T*ΔΔ*S* = +12 ± 1 kcal mol^−1^) is overwhelmed by the enthalpically favoured binding of Cu^+^ (ΔΔ*H* = −23 ± 1 kcal mol^−1^) due to the larger enthalpy of Cu^+^–thiolate bonds. These results support and quantify the proposed *in vivo* role for MT-3 in modulating the bioavailability of Cu^+^ ions.

**Table 6:** Average thermodynamic values for Zn^2+^ and Cu+ ions binding to MT-3 at pH 7.4 and 25 °C, and the difference between these thermodynamic values

Metal ion	*n*	*K*	Δ*G*° (kcal mol^−1^)	Δ*H*° (kcal mol^−1^)	−*T*Δ*S*° (kcal mol^−1^)
Zn^2+^	6.8 ± 0.3	4 (±2) × 10^11^	−15.7 ± 0.3	13.4 ± 0.2	−29 ± 0.5
Cu^+^	6 ± 1	4 (±4) × 10^19^	−27.1 ± 0.4	−10 ± 1	−17 ± 1
Difference: Cu^+^ − Zn^2+^	—	—	−11.4 ± 0.4	−23 ± 1	+12 ± 1

The presence of an unusually air stable Cu_4_^+^–thiolate cluster in the β-domain of MT-3 is thought to be important in its ability to detoxify Cu^2+^*in vivo*.^82^ The Cu^+^ clusters formed in the samples of this study differ in important ways from those formed in the reaction of MT-3 and Cu^2+^, as the binding of Cu^+^ does not require the concomitant oxidation of MT-3. However, the resulting Cu^+^_4_–thiolate cluster, which is thought to form an intriguing adamantane-like shape,^120^ must be similar, whether formed with Cu^2+^*in vivo* or by binding Cu^+^*in vitro*, given the similarities in their luminescence spectra.^82^ The approximately ten-fold difference in Cu^+^ affinity between MT-3 and MT-2 in scavenging reactions, where Cu^2+^ is reduced and bound by the protein, as measured by Calvo *et al.*,^77^ likely reflects the formation of intramolecular disulfide bonds and is the subject of ongoing studies.

## Data availability

The data supporting this article has either been included in the main body of the article or uploaded as part of the ESI.†

## Author contributions

MRM, RE, RL, RLEV, MO performed the experiments and helped analyse the data. CLV, GM, DEW, and RNA oversaw the experimental work and supervised the data analysis and drafted the manuscript. All authors reviewed and commented on multiple drafts.

## Conflicts of interest

There are no conflicts to declare.

## Supplementary Material

SC-013-D2SC00676F-s001
