# Supplementary material for: Metal binding and interdomain thermodynamics of mammalian metallothionein-3: enthalpically favoured Cu+ supplants entropically favoured Zn2+ to form Cu4+ clusters under physiological conditions
Source: Chem Sci. 2022 Apr 4;13(18):5289–304. doi: 10.1039/d2sc00676f (PMC9093145; doi:10.1039/d2sc00676f)
Supplement: SC-013-D2SC00676F-s001 [file SC-013-D2SC00676F-s001.pdf]

Electronic Supplementary Material (ESI) for Chemical Science.
This journal is © The Royal Society of Chemistry 2022

Supporting Information for

Metal Binding and Interdomain Thermodynamics of Mammalian Metallothionein-3: Enthalpically Favoured Cu^+ Supplants Entropically Favoured Zn^{2+} to form Cu^+_4 Clusters Under Physiological Conditions

Matthew R. Mehlenbacher, Rahma Elsiesy, Rabina Lakha, Rhiza Lyne E. Villones, Marina Orman, Christina L. Vizcarra, Gabriele Meloni, Dean E. Wilcox, Rachel N. Austin

Table of Contents

p. 2 Figure S1. Sequences of metallothioneins used in this study

p. 5 Figure S2. SDS-PAGE of MT-2 and MT-3 samples

p. 6 Figure S3. ITC data for DTPA → Zn^{2+} titrations in different buffers

p. 7 Figure S4. Proton plots of ITC data for DTPA → Zn_7MT-3, $Zn_4\alpha$MT-3, $Zn_3\beta$MT-3 titrations

p. 8 Figure S5. Proton plots of ITC data for $Cu^+(GSH)_2$ → Zn_7MT-3 and Zn_7MT-2 titrations

p. 8 Table S1. Best fit ITC values for DTPA → $Zn_4\alpha$MT-3 titrations

p. 8. Table S2. Best fit ITC values for DTPA → $Zn_3\beta$MT-3 titrations

p. 9. Scheme S1. Hess's Law analysis of enthalpy for DTPA → Zn_7MT-3 titrations

p. 9. Scheme S1. Hess's Law analysis of enthalpy for $Cu^+(GSH)_2$ → Zn_7MT-3 titrations

p. 10. *Post hoc* analysis for binding constant (K) of chelation ITC titrations

p. 11. Description of ITC measurements of Cu^+ → Zn_7MT-3, $Zn_4\alpha$MT-3, $Zn_3\beta$MT-3 titrations in excess MeCN

p. 12. Figure S6. ITC data for Cu^+ → Zn_7MT-3, $Zn_4\alpha$MT-3, $Zn_3\beta$MT-3 titrations in excess MeCN

Figure S1. Sequences of metallothioneins used in this study

Codons for His_6-GFP-tev-**MT-3** (based on accession # NP_038631.1):

M	G	S	S	H	H	H	H	H	H	G	S	V	S	K	G	E	E	L	F
ATG	GGC	AGC	AGC	CAT	CAT	CAT	CAT	CAT	CAC	GGA	TCC	GTG	AGC	AAG	GGC	GAG	GAG	CTG	TTC
T	G	V	V	P	I	L	V	E	L	D	G	D	V	N	G	H	K	F	S
ACC	GGG	GTG	GTG	CCC	ATC	CTG	GTC	GAG	CTG	GAC	GGC	GAC	GTA	AAC	GGC	CAC	AAG	TTC	AGC
V	S	G	E	G	E	G	D	A	T	Y	G	K	L	T	L	K	F	I	C
GTG	TCC	GGC	GAG	GGC	GAG	GGC	GAT	GCC	ACC	TAC	GGC	AAG	CTG	ACC	CTG	AAG	TTC	ATC	TGC
T	T	G	K	L	P	V	P	W	P	T	L	V	T	T	F	T	Y	G	V
ACC	ACC	GGC	AAG	CTG	CCC	GTG	CCC	TGG	CCC	ACC	CTC	GTG	ACC	ACC	TTC	ACC	TAC	GGC	GTG
Q	C	F	A	R	Y	P	D	H	M	K	Q	H	D	F	F	K	S	A	M
CAG	TGC	TTC	GCC	CGC	TAC	CCC	GAC	CAC	ATG	AAG	CAG	CAC	GAC	TTC	TTC	AAG	TCC	GCC	ATG
P	E	G	Y	V	Q	E	R	T	I	F	F	K	D	D	G	N	Y	K	T
CCC	GAA	GGC	TAC	GTC	CAG	GAG	CGC	ACC	ATC	TTC	TTC	AAG	GAC	GAC	GGC	AAC	TAC	AAG	ACC
R	A	E	V	K	F	E	G	D	T	L	V	N	R	I	E	L	K	G	I
CGC	GCC	GAG	GTG	AAG	TTC	GAG	GGC	GAC	ACC	CTG	GTG	AAC	CGC	ATC	GAG	CTG	AAG	GGC	ATC
D	F	K	E	D	G	N	I	L	G	H	K	L	E	Y	N	Y	N	S	H
GAC	TTC	AAG	GAG	GAC	GGC	AAC	ATC	CTG	GGG	CAC	AAG	CTG	GAG	TAC	AAC	TAC	AAC	AGC	CAC
K	V	Y	I	T	A	D	K	Q	K	N	G	I	K	V	N	F	K	T	R
AAG	GTC	TAT	ATC	ACC	GCC	GAC	AAG	CAG	AAG	AAC	GGC	ATC	AAG	GTG	AAC	TTC	AAG	ACC	CGC
H	N	I	E	D	G	S	V	Q	L	A	D	H	Y	Q	Q	N	T	P	I
CAC	AAC	ATC	GAG	GAC	GGC	AGC	GTG	CAG	CTC	GCC	GAC	CAC	TAC	CAG	CAG	AAC	ACC	CCC	ATC
G	D	G	P	V	L	L	P	D	N	H	Y	L	S	T	Q	S	A	L	S
GGC	GAC	GGC	CCC	GTG	CTG	CTG	CCC	GAC	AAC	CAC	TAC	CTG	AGC	ACC	CAG	TCC	GCC	CTG	AGC
K	D	P	N	E	K	R	D	H	M	V	L	L	E	F	V	T	A	A	G
AAA	GAC	CCC	AAC	GAG	AAG	CGC	GAT	CAC	ATG	GTC	CTG	CTG	GAG	TTC	GTG	ACC	GCC	GCC	GGG
																		1	2
I	T	L	G	M	D	E	L	Y	K	E	N	L	Y	F	Q	G	S	**M**	**D**
ATC	ACT	CTC	GGC	ATG	GAC	GAG	CTG	TAC	AAG	GAA	AAC	CTG	TAC	TTC	CAG	GGC	TCC	ATG	GAC
3	4	5	6	7	8	9	10	11	12	13	14	15	16	17	18	19	20	21	22
**P**	**E**	**T**	**C**	**P**	**C**	**P**	**T**	**G**	**G**	**S**	**C**	**T**	**C**	**S**	**D**	**K**	**C**	**K**	**C**
CCA	GAG	ACG	TGT	CCG	TGT	CCT	ACG	GGG	GGT	TCA	TGC	ACA	TGC	TCT	GAC	AAA	TGT	AAG	TGT
23	24	25	26	27	28	29	30	31	32	33	34	35	36	37	38	39	40	41	42
**K**	**G**	**C**	**K**	**C**	**T**	**N**	**C**	**K**	**K**	**S**	**C**	**C**	**S**	**C**	**C**	**P**	**A**	**G**	**C**
AAA	GGA	TGT	AAA	TGT	ACC	AAT	TGT	AAA	AAA	AGT	TGC	TGC	TCT	TGT	TGT	CCA	GCG	GGC	TGT
43	44	45	46	47	48	49	50	51	52	53	54	55	56	57	58	59	60	61	62
**E**	**K**	**C**	**A**	**K**	**D**	**C**	**V**	**C**	**K**	**G**	**E**	**E**	**G**	**A**	**K**	**A**	**E**	**A**	**E**
GAG	AAA	TGT	GCG	AAA	GAC	TGC	GTT	TGT	AAA	GGC	GAG	GAA	GGA	GCC	AAA	GCT	GAG	GCG	GAG
63	64	65	66	67	68														
**K**	**C**	**S**	**C**	**C**	**Q**	**.**													
AAA	TGT	TCG	TGC	TGT	CAG	TGA													

Cleaved MT-3 protein sequence used in ITC experiments:

GSMDPETCPCPTGGSCTCSDKCKCKGCKCTNCKKSCCSCCPAGCEKCAKDCVCKGEEGAKAEAEKCSCCQ

Figure S1. *continued.*

Codons for His$_6$-GFP-tev-***β*MT-3** (based on accession # NP_038631.1):

M	G	S	S	H	H	H	H	H	H	G	S	V	S	K	G	E	E	L	F
ATG	GGC	AGC	AGC	CAT	CAT	CAT	CAT	CAT	CAC	GGA	TCC	GTG	AGC	AAG	GGC	GAG	GAG	CTG	TTC
T	G	V	V	P	I	L	V	E	L	D	G	D	V	N	G	H	K	F	S
ACC	GGG	GTG	GTG	CCC	ATC	CTG	GTC	GAG	CTG	GAC	GGC	GAC	GTA	AAC	GGC	CAC	AAG	TTC	AGC
V	S	G	E	G	E	G	D	A	T	Y	G	K	L	T	L	K	F	I	C
GTG	TCC	GGC	GAG	GGC	GAG	GGC	GAT	GCC	ACC	TAC	GGC	AAG	CTG	ACC	CTG	AAG	TTC	ATC	TGC
T	T	G	K	L	P	V	P	W	P	T	L	V	T	T	F	T	Y	G	V
ACC	ACC	GGC	AAG	CTG	CCC	GTG	CCC	TGG	CCC	ACC	CTC	GTG	ACC	ACC	TTC	ACC	TAC	GGC	GTG
Q	C	F	A	R	Y	P	D	H	M	K	Q	H	D	F	F	K	S	A	M
CAG	TGC	TTC	GCC	CGC	TAC	CCC	GAC	CAC	ATG	AAG	CAG	CAC	GAC	TTC	TTC	AAG	TCC	GCC	ATG
P	E	G	Y	V	Q	E	R	T	I	F	F	K	D	D	G	N	Y	K	T
CCC	GAA	GGC	TAC	GTC	CAG	GAG	CGC	ACC	ATC	TTC	TTC	AAG	GAC	GAC	GGC	AAC	TAC	AAG	ACC
R	A	E	V	K	F	E	G	D	T	L	V	N	R	I	E	L	K	G	I
CGC	GCC	GAG	GTG	AAG	TTC	GAG	GGC	GAC	ACC	CTG	GTG	AAC	CGC	ATC	GAG	CTG	AAG	GGC	ATC
D	F	K	E	D	G	N	I	L	G	H	K	L	E	Y	N	Y	N	S	H
GAC	TTC	AAG	GAG	GAC	GGC	AAC	ATC	CTG	GGG	CAC	AAG	CTG	GAG	TAC	AAC	TAC	AAC	AGC	CAC
K	V	Y	I	T	A	D	K	Q	K	N	G	I	K	V	N	F	K	T	R
AAG	GTC	TAT	ATC	ACC	GCC	GAC	AAG	CAG	AAG	AAC	GGC	ATC	AAG	GTG	AAC	TTC	AAG	ACC	CGC
H	N	I	E	D	G	S	V	Q	L	A	D	H	Y	Q	Q	N	T	P	I
CAC	AAC	ATC	GAG	GAC	GGC	AGC	GTG	CAG	CTC	GCC	GAC	CAC	TAC	CAG	CAG	AAC	ACC	CCC	ATC
G	D	G	P	V	L	L	P	D	N	H	Y	L	S	T	Q	S	A	L	S
GGC	GAC	GGC	CCC	GTG	CTG	CTG	CCC	GAC	AAC	CAC	TAC	CTG	AGC	ACC	CAG	TCC	GCC	CTG	AGC
K	D	P	N	E	K	R	D	H	M	V	L	L	E	F	V	T	A	A	G
AAA	GAC	CCC	AAC	GAG	AAG	CGC	GAT	CAC	ATG	GTC	CTG	CTG	GAG	TTC	GTG	ACC	GCC	GCC	GGG
																		1	2
I	T	L	G	M	D	E	L	Y	K	E	N	L	Y	F	Q	G	S	**M**	**D**
ATC	ACT	CTC	GGC	ATG	GAC	GAG	CTG	TAC	AAG	GAA	AAC	CTG	TAC	TTC	CAG	GGC	TCC	ATG	GAC
3	4	5	6	7	8	9	10	11	12	13	14	15	16	17	18	19	20	21	22
**P**	**E**	**T**	**C**	**P**	**C**	**P**	**T**	**G**	**G**	**S**	**C**	**T**	**C**	**S**	**D**	**K**	**C**	**K**	**C**
CCA	GAG	ACG	TGT	CCG	TGT	CCT	ACG	GGG	GGT	TCA	TGC	ACA	TGC	TCT	GAC	AAA	TGT	AAG	TGT
23	24	25	26	27	28	29	30	31											
**K**	**G**	**C**	**K**	**C**	**T**	**N**	**C**	**K**	**.**										
AAA	GGA	TGT	AAA	TGT	ACC	AAT	TGT	AAA	TG										

Cleaved ***β***MT-3 protein sequence used in ITC experiments:

GSMDPETCPCPTGGSCTCSDKCKCKGCKCTNCK

Figure S1. *continued.*

Codons for His$_6$-GFP-tev-**αMT-3** (based on accession # NP_038631.1):

M	G	S	S	H	H	H	H	H	H	G	S	V	S	K	G	E	E	L	F
ATG	GGC	AGC	AGC	CAT	CAT	CAT	CAT	CAT	CAC	GGA	TCC	GTG	AGC	AAG	GGC	GAG	GAG	CTG	TTC
T	G	V	V	P	I	L	V	E	L	D	G	D	V	N	G	H	K	F	S
ACC	GGG	GTG	GTG	CCC	ATC	CTG	GTC	GAG	CTG	GAC	GGC	GAC	GTA	AAC	GGC	CAC	AAG	TTC	AGC
V	S	G	E	G	E	G	D	A	T	Y	G	K	L	T	L	K	F	I	C
GTG	TCC	GGC	GAG	GGC	GAG	GGC	GAT	GCC	ACC	TAC	GGC	AAG	CTG	ACC	CTG	AAG	TTC	ATC	TGC
T	T	G	K	L	P	V	P	W	P	T	L	V	T	T	F	T	Y	G	V
ACC	ACC	GGC	AAG	CTG	CCC	GTG	CCC	TGG	CCC	ACC	CTC	GTG	ACC	ACC	TTC	ACC	TAC	GGC	GTG
Q	C	F	A	R	Y	P	D	H	M	K	Q	H	D	F	F	K	S	A	M
CAG	TGC	TTC	GCC	CGC	TAC	CCC	GAC	CAC	ATG	AAG	CAG	CAC	GAC	TTC	TTC	AAG	TCC	GCC	ATG
P	E	G	Y	V	Q	E	R	T	I	F	F	K	D	D	G	N	Y	K	T
CCC	GAA	GGC	TAC	GTC	CAG	GAG	CGC	ACC	ATC	TTC	TTC	AAG	GAC	GAC	GGC	AAC	TAC	AAG	ACC
R	A	E	V	K	F	E	G	D	T	L	V	N	R	I	E	L	K	G	I
CGC	GCC	GAG	GTG	AAG	TTC	GAG	GGC	GAC	ACC	CTG	GTG	AAC	CGC	ATC	GAG	CTG	AAG	GGC	ATC
D	F	K	E	D	G	N	I	L	G	H	K	L	E	Y	N	Y	N	S	H
GAC	TTC	AAG	GAG	GAC	GGC	AAC	ATC	CTG	GGG	CAC	AAG	CTG	GAG	TAC	AAC	TAC	AAC	AGC	CAC
K	V	Y	I	T	A	D	K	Q	K	N	G	I	K	V	N	F	K	T	R
AAG	GTC	TAT	ATC	ACC	GCC	GAC	AAG	CAG	AAG	AAC	GGC	ATC	AAG	GTG	AAC	TTC	AAG	ACC	CGC
H	N	I	E	D	G	S	V	Q	L	A	D	H	Y	Q	Q	N	T	P	I
CAC	AAC	ATC	GAG	GAC	GGC	AGC	GTG	CAG	CTC	GCC	GAC	CAC	TAC	CAG	CAG	AAC	ACC	CCC	ATC
G	D	G	P	V	L	L	P	D	N	H	Y	L	S	T	Q	S	A	L	S
GGC	GAC	GGC	CCC	GTG	CTG	CTG	CCC	GAC	AAC	CAC	TAC	CTG	AGC	ACC	CAG	TCC	GCC	CTG	AGC
K	D	P	N	E	K	R	D	H	M	V	L	L	E	F	V	T	A	A	G
AAA	GAC	CCC	AAC	GAG	AAG	CGC	GAT	CAC	ATG	GTC	CTG	CTG	GAG	TTC	GTG	ACC	GCC	GCC	GGG
																		32	33
I	T	L	G	M	D	E	L	Y	K	E	N	L	Y	F	Q	G	S	**K**	**S**
ATC	ACT	CTC	GGC	ATG	GAC	GAG	CTG	TAC	AAG	GAA	AAC	CTG	TAC	TTC	CAG	GGC	TCC	AAA	AGT
34	35	36	37	38	39	40	41	42	43	44	45	46	47	48	49	50	51	52	53
**C**	**C**	**S**	**C**	**C**	**P**	**A**	**G**	**C**	**E**	**K**	**C**	**A**	**K**	**D**	**C**	**V**	**C**	**K**	**G**
TGC	TGC	TCT	TGT	TGT	CCA	GCG	GGC	TGT	GAG	AAA	TGT	GCG	AAA	GAC	TGC	GTT	TGT	AAA	GGC
54	55	56	57	58	59	60	61	62	63	64	65	66	67	68					
**E**	**E**	**G**	**A**	**K**	**A**	**E**	**A**	**E**	**K**	**C**	**S**	**C**	**C**	**Q**	.				
GAG	GAA	GGA	GCC	AAA	GCT	GAG	GCG	GAG	AAA	TGT	TCG	TGC	TGT	CAG	TGA				

Cleaved **α**MT-3 protein sequence used in ITC experiments:

GSKSCCSCCPAGCEKCAKDCVCKGEEGAKAEAEKCSCCQ

Figure S1. *continued.*

Codons for **MT-2** (based on accession # NP_005944.1):

1	2	3	4	5	6	7	8	9	10	11	12	13	14	15	16	17	18	19	20
**M**	**D**	**P**	**N**	**C**	**S**	**C**	**A**	**A**	**G**	**D**	**S**	**C**	**T**	**C**	**A**	**G**	**S**	**C**	**K**
ATG	GAC	CCG	AAC	TGC	AGC	TGC	GCG	GCG	GGT	GAC	AGC	TGC	ACC	TGC	GCG	GGC	AGC	TGC	AAG
21	22	23	24	25	26	27	28	29	30	31	32	33	34	35	36	37	38	39	40
**C**	**K**	**E**	**C**	**K**	**C**	**T**	**S**	**C**	**K**	**K**	**S**	**C**	**C**	**S**	**C**	**C**	**P**	**V**	**G**
TGC	AAA	GAG	TGC	AAG	TGC	ACC	AGC	TGC	AAG	AAA	AGC	TGC	TGC	AGC	TGC	TGC	CCG	GTG	GGT
41	42	43	44	45	46	47	48	49	50	51	52	53	54	55	56	57	58	59	60
**C**	**A**	**K**	**C**	**A**	**Q**	**G**	**C**	**I**	**C**	**K**	**G**	**A**	**S**	**D**	**K**	**C**	**S**	**C**	**C**
TGC	GCG	AAA	TGC	GCG	CAG	GGT	TGC	ATC	TGC	AAG	GGC	GCG	AGC	GAT	AAA	TGC	AGC	TGC	TGC
61																			
**A**	**.**																		
GCG	TAA																		

MT-2 protein sequence used in ITC experiments:

MDPNCSCAAGDSCTCAGSCKCKECKCTSCKKSCCSCCPVGCAKCAQGCICKGASDKCSCCA

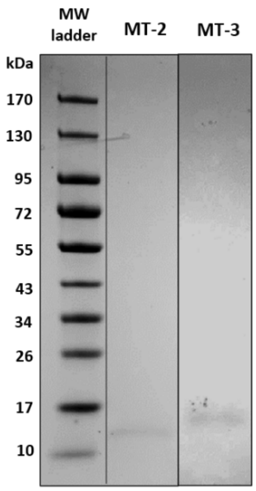

Figure S2. SDS-PAGE of fractions containing MT-2 and MT-3 obtained upon the reaction of Cu^{+} with Zn_7MTs and subsequent MT separation by Size Exclusion Chromatography (SEC). The MT Cys residues were modified with monobromobimane (mBrB) prior to SDS-PAGE and the protein bands were stained with Coomassie Blue.

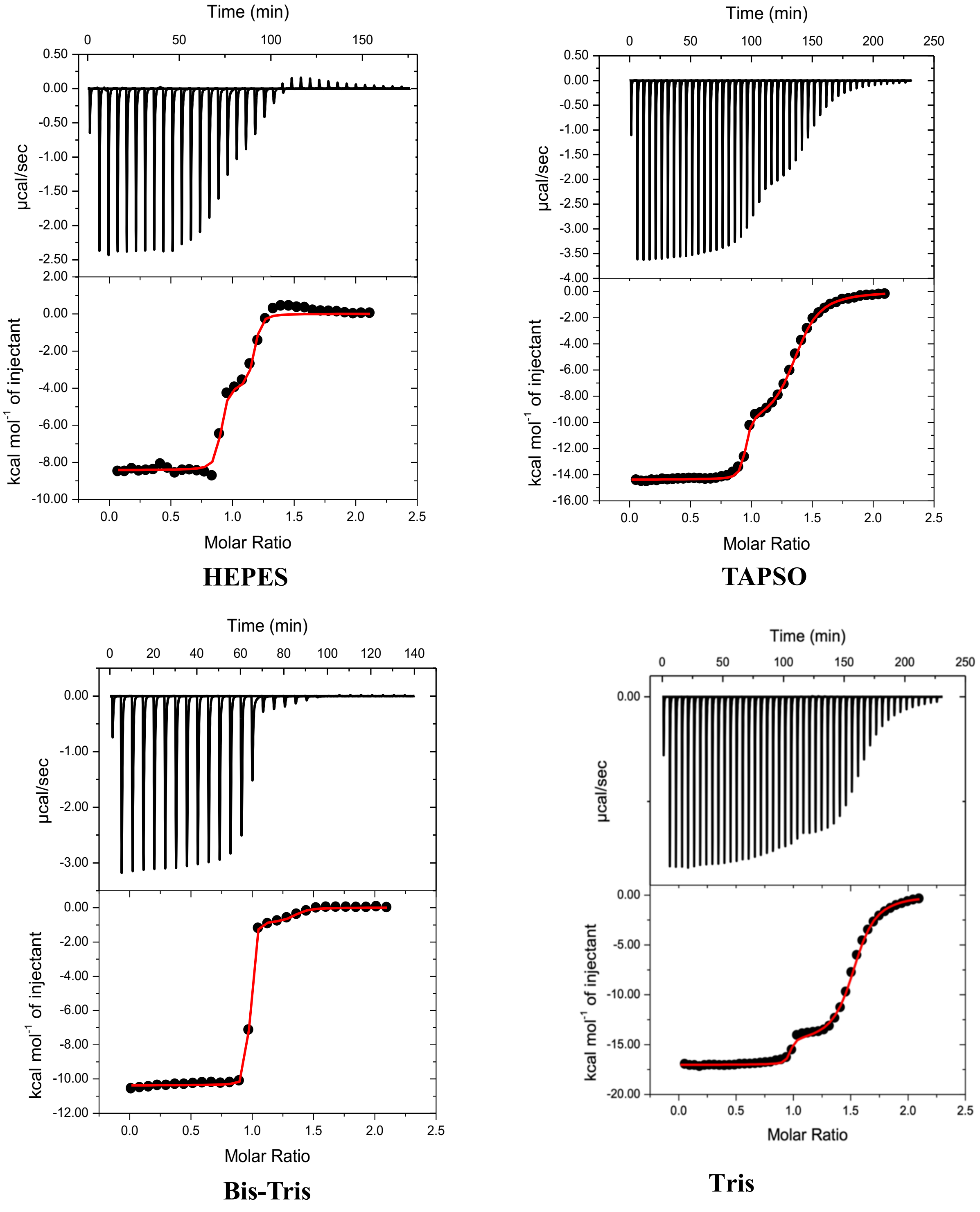

Figure S3. Representative thermograms for DTPA titrations into Zn^{2+} in HEPES, Bis-Tris, TAPSO and Tris buffers (100 mM buffer, 150 mM NaCl, pH 7.4); fits to a 2-sites binding model give n = 1 for the first event, formation of the 1:1 DTPA-Zn^{2+} species, followed by an event with n ~ 0.5, corresponding to the subsequent formation of the 3:2 DTPA:Zn^{2+} species

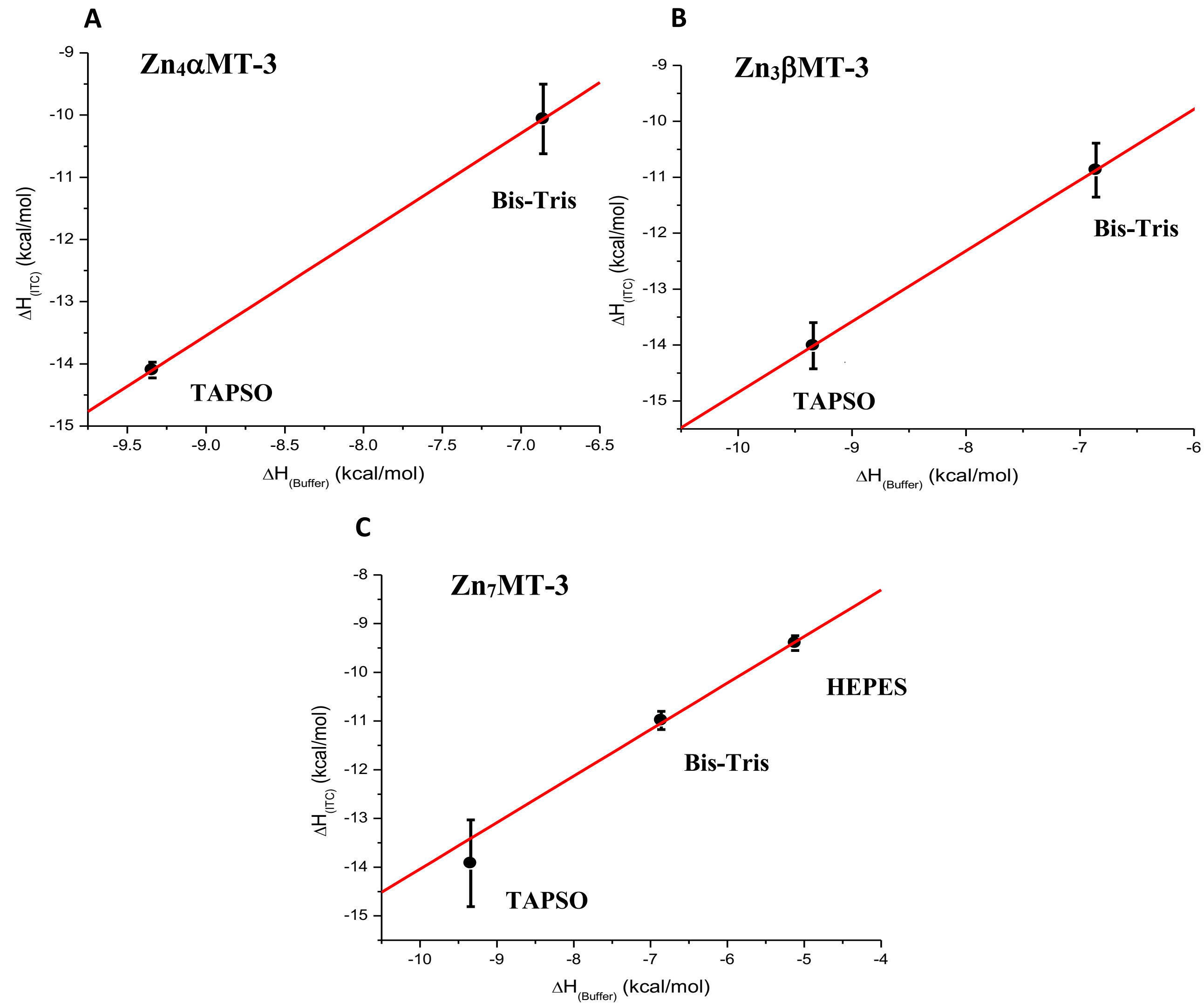

Figure S4. Plots of the experimental enthalpy (ΔH_{ITC}) versus the buffer protonation enthalpy (ΔH_{buffer}) in 2-3 different buffers for DTPA chelation of Zn^{2+} from **A.** $Zn_4\alpha$MT-3, slope = +1.6 ± 0.3, **B.** $Zn_3\beta$MT-3, slope = +1.3 ± 0.3, and **C.** Zn_7MT-3, slope = +1.0 ± 0.1, where the slope quantifies the number of protons binding to, or released from, the buffer.

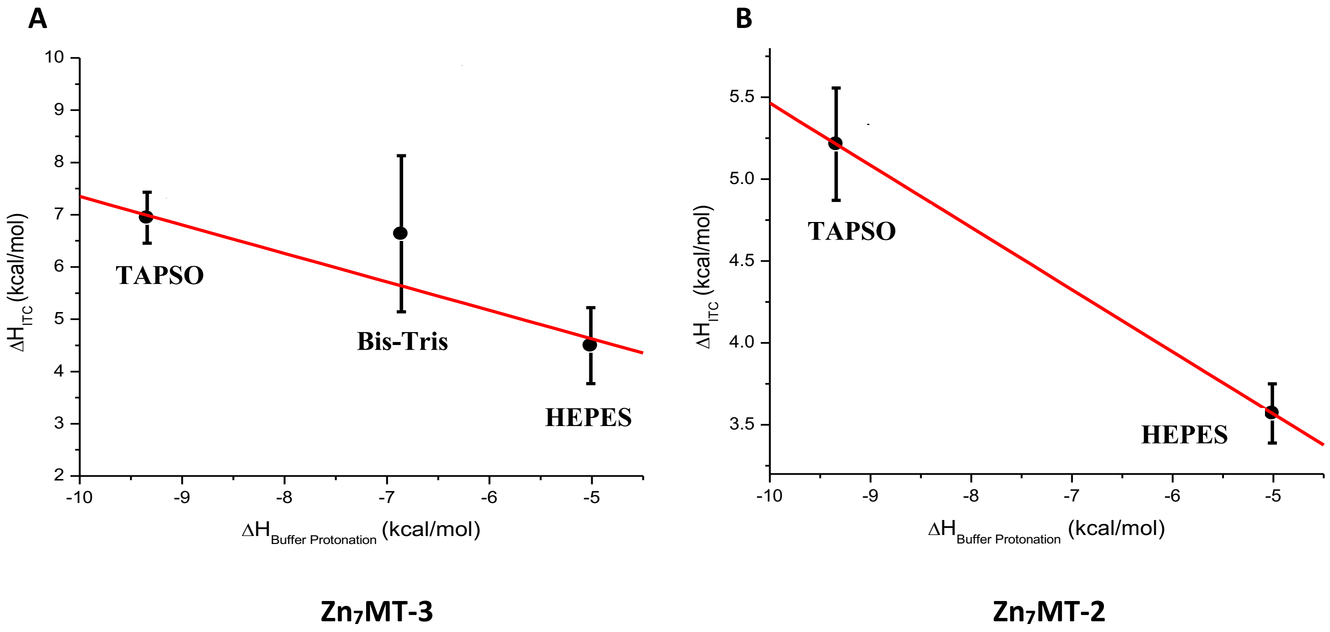

Figure S5. Plots of the experimental enthalpy (ΔH_{ITC}) versus the buffer protonation enthalpy (ΔH_{buffer}) in 2-3 different buffers for Cu^+ titrations into Zn_7MT in excess GSH; **A.** Zn_7MT-3, slope = −0.5 ± 0.2 and **B.** Zn_7MT-2, slope = −0.4 ± 0.1, where the slope quantifies the number of protons binding to, or released from, the buffer.

Table S1. Average best-fit experimental values for DTPA chelation of Zn^{2+} from $Zn_4\alpha$MT-3 obtained from fits of the first binding event, which is the chelation of Zn^{2+} from the protein.

buffer	n_{ITC}	K_{ITC}	ΔH^o_{ITC} (kcal/mol)
Bis-Tris	4.4 ± 0.3	9 (± 5) X 10^7	−10.1 ± 0.6
TAPSO	4.2 ± 0.2	6.5 (± 0.3) X 10^6	−14.1 ± 0.1

Table S2. Average best-fit experimental values for DTPA chelation of Zn^{2+} from $Zn_3\beta$MT-3 obtained from fits of the first binding event, which is the chelation of Zn^{2+} from the protein.

buffer	n_{ITC}	K_{ITC}	ΔH^o_{ITC} (kcal/mol)
Bis-Tris	2.6 ± 0.1	2.8 (± 0.6) X 10^7	−10.9 ± 0.5
TAPSO	2.8 ± 0.2	4 (± 1) X 10^6	−14.1 ± 0.4

Scheme S1. Relevant equilibria for DTPA chelation of Zn^{2+} from Zn_7MT-3 at pH 7.4. DTPA has two relevant pK_a's: 8.60, which provides 0.91 H^+, and 10.55, which provides 1 H^+ (13.4 H^+ released from 7 DTPA upon Zn^{2+} binding at pH 7.4) ΔH_{ML} is the enthalpy of the metal (M) – ligand (L) interaction, ΔH_{LH1} and ΔH_{LH2} are the enthalpies of deprotonation of the ligand (H_1 and H_2), ΔH_{PH} is the enthalpy of the protonation of the protein (P), ΔH_{BH} is the enthalpy of buffer (B) protonation, and ΔH_{MP} is the enthalpy of the desired metal-protein interaction.

$$7\ DTPAH^{+}_{1.91} + Zn^{2+}{}_{7}MT\text{-}3 + (13.4\text{-}X)\ Buffer \leftrightarrows 7\ Zn^{2+}DTPA + MT\text{-}3H^{+}_{X} + (13.4\text{-}X)\ BufferH^{+}$$

$DTPA + Zn^{2+} \leftrightarrows Zn^{2+}DTPA$	$7 \times \Delta H_{ML}$
$DTPAH^{+}_{1.0} \leftrightarrows DTPA + H^{+}$	$-7 \times \Delta H_{LH1}$
$DTPAH^{+}_{0.91} \leftrightarrows DTPA + 0.91\ H^{+}$	$-6.4 \times \Delta H_{LH2}$
$MT\text{-}3 + H^{+} \leftrightarrows MT\text{-}3H^{+}$	$X \times \Delta H_{PH}$
$B + H^{+} \leftrightarrows BH^{+}$	$(13.4 - X) \times \Delta H_{BH}$
$Zn^{2+}{}_{7}MT\text{-}3 \leftrightarrows MT\text{-}3 + 7\ Zn^{2+}$	$-7 \times \Delta H_{MP}$

$$\Delta H_{ITC} = 7\Delta H_{ML} - 7(\Delta H_{LH1}) - 6.37(\Delta H_{LH2}) + (13.4 - X)\ \Delta H_{BH} + X(\Delta H_{PH}) - 7\Delta H_{MP}$$

Scheme S2. Relevant equilibria for Cu^+ binding to MT-3 in the presence of excess GSH at pH 7.4. Each GSH picks up 0.8 H^+ when released from the $Cu^+(GSH)_2$ complex (9.6 H^+ bind to 12 GSH upon release from Cu^+ at pH 7.4). ΔH_{ML} is the enthalpy of the metal (M) – ligand (L) interaction, ΔH_{LH} is the enthalpy of protonation of the ligand, ΔH_{PH} is the enthalpy of the protonation of the protein (P), ΔH_{BH} is the enthalpy of buffer (B) protonation, and ΔH_{MP} is the enthalpy of the desired metal-protein interaction.

$$6\ Cu^{+}(GSH)_2 + MT\text{-}3H^{+}_{X} + (9.6\text{-}X)\ BufferH^{+} \leftrightarrows Cu^{+}{}_{6}MT\text{-}3 + 12\ GSH^{+}_{0.8} + (9.6\text{-}X)\ Buffer$$

$Cu^{+}(GSH)_2 \leftrightarrows Cu^{+} + GSH$	$-6 \times \Delta H_{ML}$
$GSH + 0.8\ H^{+} \leftrightarrows GSH^{+}_{0.8}$	$9.6 \times \Delta H_{LH}$
$MT\text{-}3H^{+} \leftrightarrows MT\text{-}3 + H^{+}$	$-X \times \Delta H_{PH}$
$BH^{+} \leftrightarrows B + H^{+}$	$-(9.6 - X) \times \Delta H_{BH}$
$6\ Cu^{+} + MT\text{-}3 \leftrightarrows Cu^{+}{}_{6}MT\text{-}3$	$6 \times \Delta H_{MP}$

$$\Delta H_{ITC} = -6\Delta H_{ML} + 9.6(\Delta H_{LH}) - (9.6 - X)\ \Delta H_{BH} - X(\Delta H_{PH}) + 6\Delta H_{MP}$$

Post-hoc Analysis to Determine the Binding Constant from Chelation Measurements

Consider the following experimental equilibrium constant for the chelation of a metal (M) from the protein (P) using ligand (L) (e.g., L + MP → P + ML):

$$K_{ITC} = \frac{[ML]}{[M]_{ITC}[L]_{ITC}} \qquad Eq. 1$$

where $[M]_{ITC}$ and $[L]_{ITC}$ are the concentrations of all metal and ligand species, respectively, except the ML complex. The total concentrations of the metal (C_M) and the ligand (C_L), where two protonation states are considered for the ligand, are:

$$C_M = [M] + [MP] + [ML] \qquad Eq. 2$$

$$C_L = [L] + [L_{H1}] + [L_{H2}] + [ML] \qquad Eq. 3$$

Each species has an equilibrium formation constant defined in Eqs. 4, 5, 6, and 7:

$$K_{MP} = \frac{[MP]}{[M][P]} \qquad Eq. 4$$

$$K_{ML} = \frac{[ML]}{[M][L]} \qquad Eq. 5$$

$$K_{L_{H1}} = \frac{[LH]}{[L][H]} \qquad Eq. 6$$

$$K_{L_{H2}} = \frac{[LH_2]}{[LH][H]} \qquad Eq. 7$$

Equation 8 can be introduced:

$$K_{L_{H1}}\, x\, K_{L_{H2}} = \beta_{LH} \qquad Eq. 8$$

We now consider the species included in $[M_{ITC}]$ and $[L_{ITC}]$:

$$[M_{ITC}] = C_M - [ML] = [M] + [MP] = [M] + K_{MP}[M][P] = [M](1 + K_{MP}[P]) \qquad Eq. 9$$

$$[L_{ITC}] = C_L - [ML] = [L] + [L_{H1}] + [L_{H2}] = [L](1 + K_{LH1}[H^+] + \beta_{LH}[H^+]^2) \qquad Eq. 10$$

Substituting these into the original experimental equilibrium expression (*Eq.* 1):

$$K_{ITC} = \frac{[ML]}{[M][L]} \, X \, \frac{1}{(1 + K_{MP}[P])} \, X \, \frac{1}{(1 + K_{LH1}[H^+] + \beta_{LH}[H^+]^2)} \qquad Eq.11$$

Finally, this equation can be rearranged to solve for the metal-protein equilibrium constant:

$$K_{MP} = \left(\frac{K_{ML}}{K_{ITC}\,(1 + K_{LH1}[H^+] + \beta_{LH}[H^+]^2)} - 1\right) \Big/ [P] \qquad Eq.12$$

The equilibrium constant, K_{MP}, from the chelation of Zn^{2+} from MT-3 with DTPA, however, does not take into account the competition from protonation on a *per-metal* basis, but on a *per-protein* basis. The former is determined by first solving for the K_{MP} on a *per-protein* basis by multiplying the K_{ITC} and K_{ML} by the stoichiometry, n_{ITC}. Other values, such as $[H^+]$, for example, remain constant. The resulting *per-protein* equilibrium constant is then divided by the experimental stoichiometry to determine the proton-corrected *per-metal* equilibrium constant.

Cu^+ Binding to MT-3 and Its α and β Domains in Excess Acetonitrile (MeCN)

Studies of Cu^+ binding to proteins often introduce this metal ion from anaerobic aqueous solutions prepared with $[Cu(MeCN)_4]PF_6$ and excess MeCN, which stabilizes the Cu^+ from disproportionation to Cu^{2+} and Cu^0. These solutions contain predominantly $Cu^+(MeCN)_{3(aq)}$ (log β_3 = 4.23), as $Cu^+(MeCN)_{4(aq)}$ is only marginally more stable.[1] To correlate our ITC measurements of Cu^+ binding to MT with previous studies that used MeCN to stabilize the Cu^+, especially measurements on MT-1a that characterized the species with ESI-MS,[2] we titrated Cu^+ solutions that were stabilized by a 10-fold excess of MeCN into solutions of Zn_7MT-3 and its α and β domains. Representative data for these ITC measurements are shown in Figure S6.

Due to several unknown competing equilibria (for example Zn^{2+}-MeCN) in these titrations, as well as concern about the impact of the large excess of MeCN on the protein in the cell, which is required for the ITC measurements, quantitative analysis of the Cu^+ binding constants and thermodynamics is not possible or prudent. However, the titration isotherms do provide evidence for different populations of Cu^+ binding to MT-3 with the weakly competing Cu^+-stabilizing ligand MeCN. The titration of Cu^+ into MT-3 shows three distinct binding events, an initial subtle one (see insert) at an approximate stoichiometry of 8 Cu^+, a second at 10 Cu^+, and a third at 20 Cu^+. The titration of Cu^+ into $Zn_3\beta$MT3 also shows 3 binding events, the first with a stoichiometry of 2 Cu^+ and a significantly greater exothermic enthalpy, followed by a second at 4 Cu^+ and a third at approximately 9 Cu^+. Finally, the titration of Cu^+ into $Zn_4\alpha$MT3 shows 2 binding events, the first at a stoichiometry of 6 Cu^+, followed by a second at 11 Cu^+ . In all three cases, the final stoichiometry matches a 1:1 ratio of Cu^+-to-Cys in the protein, as found by ESI-MS on samples prepared with a similar addition of Cu^+ in excess MeCN.

These results contrast with those found when physiologically relevant GSH is used to stabilize the Cu^+ and it competes with MT for the Zn^{2+}. The ITC data with MeCN reflect Cu^+ displacement of Zn^{2+} from the protein and the eventual formation of species with Cu^+ bound to each Cys. The presence of GSH represents a situation more relevant to *in vivo* conditions.

Nevertheless, the ITC data with MeCN provide corroborating evidence for initial, and more tightly bound, Cu^+ in Cu^+_4 clusters in the β domain and the α domain, as indicated by Cu^+_8MT-3.

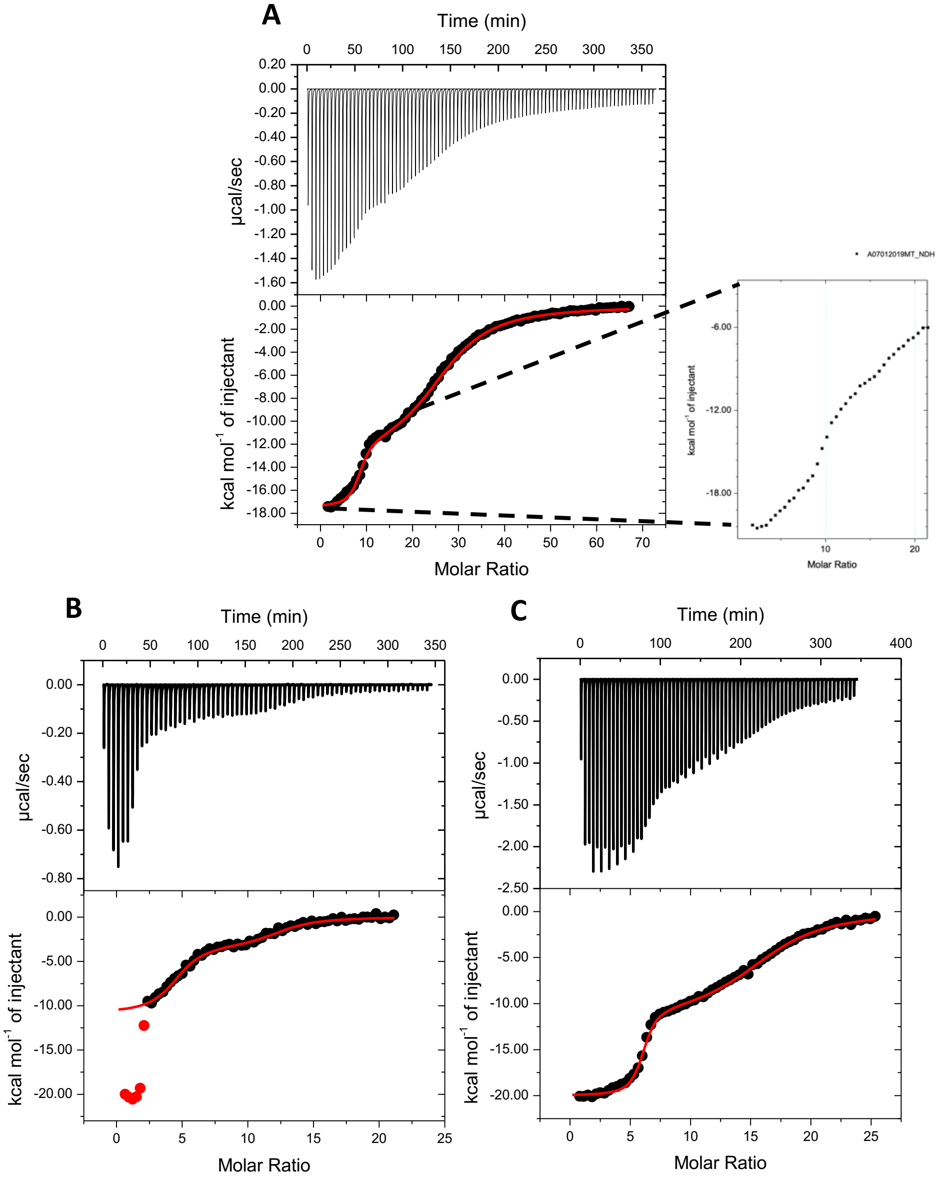

Figure S6. Representative thermograms for Cu^+ titrated into **A.** Zn_7MT-3, **B.** $Zn_3\beta$MT-3, **C.** $Zn_4\alpha$MT-3 in 100 mM Bis-Tris, 150 mM NaCl, 6 mM MeCN, pH 7.4; fitting the isotherms gives approximate binding stoichiometries for **A**: $n_1 = 8$, $n_2 = 10$, $n_3 = 20$; **B**: $n_1 = 2$, $n_2 = 4$, $n_3 = 9$; **C**: $n_1 = 6$ and $n_2 = 11$.

1. Martell, A. E.; Smith, R. K. Critical Stability Constants, Standard Reference Database 46. National Institute of Standards and Technology: Gaithersburg, MD 2001.

2. Scheller, J. S.; Irvine, G. W.; Wong, D. L.; Hartwig, A.; Stillman, M. J. Stepwise Copper(I) Binding to Metallothionein a Mixed Cooperative and Non-Cooperative Mechanism for All 20 Copper Ions. *Metallomics* **2017**, *9* (5), 447–462.
